# Deep longitudinal multi-omics analysis of *Bordetella pertussis* cultivated in bioreactors highlights medium starvations and transitory metabolisms, associated to vaccine antigen biosynthesis variations and global virulence regulation

**DOI:** 10.3389/fmicb.2023.1036386

**Published:** 2023-02-14

**Authors:** Paul Anziani, Jérémie Becker, Charlotte Mignon, Nadège Arnaud-Barbe, Virginie Courtois, Marie Izac, Romain Pizzato, Joséphine Abi-Ghanem, Viet-Dung Tran, Magali Sarafian, Andrei Bunescu, Dominique Garnier, Eric Abachin, Geneviève Renauld-Mongénie, Cyril Guyard

**Affiliations:** ^1^Sanofi, Marcy-l’Étoile, France; ^2^BIOASTER, Lyon, France

**Keywords:** *Bordetella pertussis*, bacterial culture, virulence, antigen production, longitudinal multi-omics, fermentation molecular profiling, cysteine starvation, proline starvation

## Abstract

*Bordetella pertussis* is the bacterial causative agent of whooping cough, a serious respiratory illness. An extensive knowledge on its virulence regulation and metabolism is a key factor to ensure pertussis vaccine manufacturing process robustness. The aim of this study was to refine our comprehension of *B*. *pertussis* physiology during *in vitro* cultures in bioreactors. A longitudinal multi-omics analysis was carried out over 26 h small-scale cultures of *B*. *pertussis*. Cultures were performed in batch mode and under culture conditions intending to mimic industrial processes. Putative cysteine and proline starvations were, respectively, observed at the beginning of the exponential phase (from 4 to 8 h) and during the exponential phase (18 h 45 min). As revealed by multi-omics analyses, the proline starvation induced major molecular changes, including a transient metabolism with internal stock consumption. In the meantime, growth and specific total PT, PRN, and Fim2 antigen productions were negatively affected. Interestingly, the master virulence-regulating two-component system of *B*. *pertussis* (BvgASR) was not evidenced as the sole virulence regulator in this *in vitro* growth condition. Indeed, novel intermediate regulators were identified as putatively involved in the expression of some virulence-activated genes (*vags*). Such longitudinal multi-omics analysis applied to *B*. *pertussis* culture process emerges as a powerful tool for characterization and incremental optimization of vaccine antigen production.

## Introduction

*Bordetella pertussis* is the bacterial causative agent of pertussis or whooping cough. Despite widespread vaccination programs, *B*. *pertussis* is still in circulation with an estimated 24 million cases and 160,000 deaths in children younger than 5 years in 2014 (Yeung et al., 2017). To reduce this public health burden, two major types of vaccine are in use. The first one is a whole-cell pertussis vaccine (wP) composed of inactivated *B*. *pertussis*. The second is an acellular pertussis vaccine (aP), composed of purified *B*. *pertussis* virulence factors. The most commonly purified antigens found in aP are the pertussis toxin (PT) and the filamentous hemagglutinin (FHA). Some aPs are supplemented with pertactin (PRN) and fimbriae (Fim; Edwards and Decker, 2018). Vaccine developers continuously work on process optimization of wP and aP vaccines, especially antigen production during *B*. *pertussis* culture (Dehottay and Goffin, 2013; Dehottay et al., 2015). Despite their critical roles during the production of pertussis vaccines, the biological mechanisms and metabolic pathways, which take place during culture process, are not fully understood.

In 2004, the FDA launched the process analytical technology (PAT) initiative, which aims at improving the robustness and consistency of pharmaceutical manufacturing processes. PAT provides a strategy to obtain maximum control of product and process based upon scientific understanding (Food and Drug Administration, 2004). To this end, with a focus on ensuring the robustness of their production, vaccine manufacturers are constantly facing new challenges. As any other bioproduction processes, industrial antigen production is at risk of being hampered by low yield and batch failure. In addition, vaccine producers face a fluctuating demand for their products and must provide consistent scale-up and scale-down processes. Moreover, health authorities recommend the suppression of raw material from animal and, more recently, non-chemically defined supplements (Food and Drug Administration, 2019). To incrementally adapt to these challenges, access to an extensive knowledge of *B*. *pertussis* physiology is a prerequisite, particularly regarding the general metabolism and the molecular regulatory mechanisms associated with vaccine antigen production.

*Bordetella pertussis* displays several defective metabolic pathways. It is reported to have dysfunctional glycolysis and requires niacin supplementation for growth. *Bordetella pertussis* only uses cysteine as sulfur source and its tricarboxylic acid (TCA) cycle has long been considered partially non-functional (Jebb and Tomlinson, 1957; Thalen et al., 1999; Parkhill et al., 2003). However, *B*. *pertussis* TCA cycle was recently shown to be entirely functional in the Tohama I strain using chemically defined SS-medium (Izac et al., 2015). In addition, some *B*. *pertussis* strains are also able to use thiosulfate as a sulfur source. Thus, *B*. *pertussis* metabolic capabilities are strain-dependent (Branco dos Santos et al., 2017).

The regulation of *B*. *pertussis* virulence during bacterial culture has been well documented over the past decades. Several studies uncovered the regulatory mechanisms of the main virulence regulon BvgASR in specific *B*. *pertussis* strains, using virulence modulators such as niacin or MgSO_4_ in flask culture models followed by transcriptomic analyses (Hot et al., 2003; Moon et al., 2017; Coutte et al., 2020). Recently, two additional two-component systems, RisAK and PlrSR, were shown to be involved in *B*. *pertussis* virulence regulation, *in vitro* and *in vivo*, respectively (Cróinín et al., 2005; Coutte et al., 2016; Bone et al., 2017). Despite these findings which were mostly obtained using flask culture models, a lot remains to be uncovered to have a global picture of *B*. *pertussis* metabolism and to fully understand *B*. *pertussis* virulence regulation during culture.

With the development of OMICs technologies, deep molecular longitudinal analyses of culture processes are now accessible, allowing the monitoring of thousands of molecules interacting across different omics. Such multi-omics approaches have been successfully applied in a wide range of biological fields including microbiology (Hasin et al., 2017; O’Donnell et al., 2020). Through a study combining proteomic and microarray analyses on a chemostat model, proteomics was shown to be a valuable method to monitor and assess the quality of *B*. *pertussis* antigens along the wP upstream production process (Metz et al., 2017). In a subsequent study, a genome-scale metabolic model combined to an end-point extracellular metabolomic analysis provided a new insight into the metabolic capabilities of *B*. *pertussis* and led to a > 2-fold improvements in pertussis toxin production (Branco dos Santos et al., 2017). More recently, the combination of longitudinal and multi-omics analyses demonstrated its value for the characterization and optimization of industrial culture bioprocesses as shown with the fungus *Aspergillus niger* (Lu et al., 2018) or with the bacterium *Zymomonas mobilis* (Zhang et al., 2019). Despite initial very promising investigations (Nakamura et al., 2006; Van De Waterbeemd et al., 2009; Branco dos Santos et al., 2017), such an extensive longitudinal multi-omics study has never been reported before on *B*. *pertussis* using experimental conditions mimicking industrial culture processes.

Here, we report the first longitudinal investigation of a small-scale *B*. *pertussis* bioreactor model combining transcriptomic, proteomic, lipidomic, and metabolomic analyses. Data obtained with this model that intends to mimic industrial process, provide an insight into the metabolism and the regulation networks of *B*. *pertussis* during culture in bioreactors.

## Materials and methods

### Bacterial strain

A colony of *B*. *pertussis* Tohama I (ATCC BAA-589) was isolated from a lyophilized commercial stock solution cultured on a Bordet Gengou agar (BD, #254400) plate. From this colony, a master seed bank was obtained using a 48 h cultivation on Bordet Gengou agar plate and aliquoted in Bovine Serum Albumin—Saccharose Phosphate Glutamate buffer. A working seed bank was next produced from the master seed bank cultured using a chemically defined SS-medium in a fermenter. The culture was then harvested in exponential growth phase and aliquoted following the addition of 20% of glycerol and stored at −80°C.

### Bacterial culture

**Figure 1:**
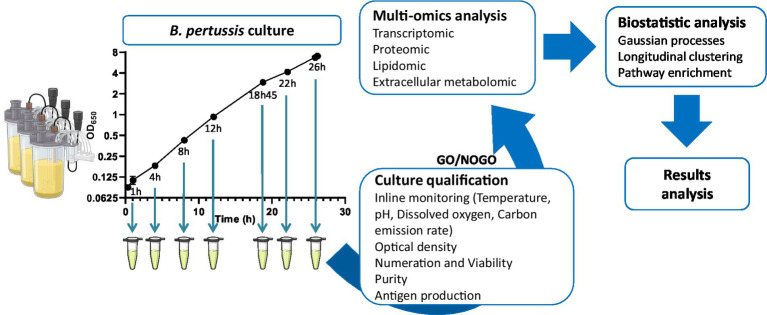
Experimental plan of the longitudinal multi-omics study of *Bordetella pertussis* culture.

Six batch cultures of *Bordetella pertussis* (Figure 1), strain Tohama I (ATCC®BAA-589), were performed in a chemically defined medium derived from the Stainer-Scholte (SS) medium as previously described (Izac et al., 2015). This medium reproduces the composition of casein hydrolysate-containing medium used in industrial processes (Imaizumi et al., 1983; Quentin-Millet et al., 1987). Preculture and cultures were carried out in small scale biofermenters (Ambr® 250, Sartorius), in a batch mode at 36°C, pH 7.3, and 26% of dissolved oxygen (DO). Anti-foam C (Dow Corning™) was used to control foaming and pH was regulated by automatic addition of 10% (v/v) H_3_PO_4_. Dissolved oxygen was controlled by a regulation cascade of stirring (350–1,300 rpm), air injection (10–110 ml.min^−1^), and dioxygen injection (0–80 ml.min^−1^).

Briefly, preculture was inoculated from the frozen working seed bank. After 22 h of growth, an appropriate volume of preculture was centrifuged to replace preculture supernatant with fresh medium. Fermenters were inoculated at 0.1 of optical density at 650 nm wavelength (OD_650_). Sampling was performed for quality control (QC) testing, antigen quantification, and omics analyses. End of the cultures were determined by a sudden decrease of carbon emission rate (CER), and an increase of dissolved oxygen.

### Culture quality control

During cultures, temperature, pH, dissolved oxygen, foaming, and CER were monitored using the Ambr® 250 platform. Growth monitoring was ensured by measuring the OD_650_ (V-630 Spectrophotometer, Jasco®) and flow cytometry viability assays. Numeration and bacterial viability were analyzed using a Guava® easyCyte™ flow cytometer (Luminex) with Live/Dead® BacLight™ kit (Thermo Fisher Scientific). At the end of cultures, samples were plated on tryptic soy agar (TSA; BD, #254086) plate to control the culture purity and the absence of avirulent phenotype.

### Vaccine antigens quantification

The quantification of vaccine antigens was performed at 1 h, 4 h, 8 h, 12 h, 18 h 45 min, 22 h, and 26 h of culture by enzyme-linked immunosorbent assay (ELISA; Figure 1). At the end of the cultures, antigen concentrations were measured in triplicate for each culture. Run acceptance criteria was defined, for all final antigen concentrations, at CV < 15% between bioreactors.

Briefly, 125 μl of crude harvest was lysed by incubation with 50% v/v of lysis buffer (Xtractor™buffer, Clontech) during 20 min at room temperature and stored at −80°C. The lysis solution contained 20 U.ml^-1^ of Turbo DNase (Thermo Fisher, #AM2239), 0.4 mg.ml^−1^ of lysozyme (Sigma-Aldrich, #L3790), and ethylenediaminetetraacetic acid (EDTA)-free protease inhibitor cocktail (cOmplete™, Roche). Antigen quantifications were performed by sandwich ELISA on *B*. *pertussis* lysed suspension using proprietary monoclonal antibodies against specific proteins: PT, FHA, PRN, and Fimbriae 2 (Fim2). Briefly, 96 well plates were coated with monoclonal antibodies anti-PT, anti-FHA, anti-PRN, or anti-Fim2, at a concentration of 3, 2, 2, and 2.5 μg.ml^−1^, respectively. After a saturation step, samples were diluted to appropriate concentration. After incubation and wash steps, plates were incubated with biotinylated secondary antibodies anti-PT, anti-FHA, anti-PRN or anti-Fim2. Then, a solution of streptavidine-HRP (Jackson Immunoresearch) was added to the plates. Plates were revealed with tetramethylbenzidine (Tebu-bio) incubation during 20 min at room temperature. The reaction was stopped by the addition of 1 N HCl. Optical density at 450 nm (OD_450_) and 620 nm (OD_620_) wavelengths were measured using a microplate reader PHERAstar™ FS (BMG Labtech). OD_620_ values were subtracted to OD_450_ to consider plastic absorption. Antigen concentrations were determined using SoftMax® Pro (v6.5) software (Molecular Devices) with a standard curve obtained using purified antigens as reference standards.

### Sampling methods for multi-omics analyses

A total of six batch cultures were used in this study. Three of the six cultures were used to collect samples after 1 h of culture. Due to the low level of biomass at the beginning of the culture, large volumes of the bacterial cultures had to be collected to allow multi-omics analyses. Thus, to prevent a bias due to a change of the culture conditions in the subsequent time points, those three bioreactors were stopped. The three remaining bioreactors were sampled after 4 h, 8 h, 12 h, 18 h 45 min, 22 h and 26 h of culture for all omics analyses (Figure 1).

For transcriptomic analyses, a volume corresponding to 1 OD_650_ in 1 ml (1 ODu) was sampled and incubated for 1 h at room temperature with 33% v/v of RNAlater (Invitrogen). Then, samples were centrifuged for 10 min at 4,000 *g* at 4°C and supernatants were discarded.

For proteomic analyses, 1 ODu was sampled and centrifuged for 10 min at 4,000 *g* at 4°C. Supernatants were eliminated, and pellets were washed twice with 5 ml of cold PBS.

For lipidomic analyses, 4 ODu were sampled and mixed with 50% (v/v) of quenching solution (40% ethanol/0.8% NaCl at −25°C; Spura et al., 2009).Then, samples were immediately centrifuged for 10 min at 4,000 *g* and 4°C and supernatants were eliminated.

For extracellular metabolomic analyses, at least 300 μl were sampled, centrifuged for 10 min at 4,000 *g* at 4°C and supernatants were collected.

For all omics analyses, samples were immediately frozen in dry ice and stored at −80°C until analysis.

### Transcriptomic data generation and analysis

Bacteria pellets were lysed in 200 μl of Tris-EDTA buffer (Invitrogen™) with 15 mg.ml^−1^ of lysozyme (Thermo Fisher Scientific) and 1 mg.ml^−1^ of proteinase K (Qiagen) for 10 min at 400 rpm, at room temperature. Then, RNA extraction was performed on column using RNeasy® mini kit (Qiagen) including an on-column DNase digestion. A second step of DNase digestion was performed by DNase TURBO™ (Thermo Fisher Scientific). RNA purity was assessed using NanoDrop™ 2000 (Thermo Scientific™). RNA integrity was analyzed by TapeStation 4200 (Agilent) using the High Sensitivity RNA ScreenTape. Only samples with a RINe over 7 were selected. RNA was quantified using Qubit™ 2.0 Fluorometer (Thermo Fisher Scientific) with the Qubit™ RNA high sensitivity kit (Invitrogen™).

Libraries were prepared in triplicate for each sample with the Sciclone® G3 NGSx iQ™ liquid handling workstation (PerkinElmer) by using the Universal RNA-Seq kit (Tecan Genomics, 2021) customized for *B*. *pertussis*. Briefly, cDNA was synthetized by reverse transcription and mechanically fragmented with the Covaris® M220 Focused-ultrasonicator™ for 90 s at 50 W, 10% duty factor, and 200 burst per cycle, to obtain 400 bp (base pair) cDNA fragments. Fragments were, then, purified using Agencourt® beads, followed by end repair, adaptor ligation, and sense strand selection steps. Ribosomal RNAs (rRNA) were depleted using AnyDeplete technology (Tecan Genomics) with specific *B*. *pertussis* rRNA probes. Libraries obtained after PCR amplification (18 cycles) were characterized by TapeStation with D5000 ScreenTape (Agilent), normalized at 10 nM, pooled, diluted at 4 nM and, denatured according to Illumina NextSeq500 protocol A (Illumina, 2022). Denatured libraries were diluted at 1.8 pM and 1 μl of PhiX at 20 pM was added as control. Then, three sequencing runs were launched, with 1.3 ml of this mix on a NextSeq® 500 platform (Illumina) using 75-bases single-end protocol on a High Output flow cell (Illumina).

Following base calling steps by sequencer software (Illumina), quality control of the sequencing run was carried out using Sequencing Analysis Viewer (SAV, Illumina, version 2.4.7) regarding reads quality and cluster density. After demultiplexing steps, sequencing QC was performed on raw data with fastqc tool (Babraham Bioinformatics, 2019) and visualized using multiQC tool (Ewels et al., 2016).

Raw data were imported in Array Studio (v11.0) software (OmicSoft Qiagen). Reads were trimmed to remove low quality bases with default parameters and mapped to the NCBI genome of the strain of *B*. *pertussis* Tohama I (RefSeq accession number NC_002929.2; Parkhill et al., 2003). Finally, QC was performed on aligned reads. Count table was generated with default parameters. Transcripts were normalized using RLE (implemented in DESeq2 R package; Love et al., 2014), those with low abundance (mean normalized expression under 10) were filtered out.

### Proteomic data generation and analysis

Due to technical limitations, simultaneous analyses of the secretome and cellular proteome could not be achieved in this study. Proteomic analyses were thus solely performed on cellular proteome. Frozen cell pellets were thawed by the addition of 130 μl lysis buffer [4% SDS, 50 mM Tris–HCl (pH 7.5)]. After resuspension, the bacteria lysates were boiled at 95°C on a thermoshaker for 10 min. Following cooling at room temperature, lysates were transferred to Covaris tubes and subjected to ultrasonication on a Covaris® M220 Focused-ultrasonicator™ for 90 s at 75 W, 20% duty factor, and 200 burst per cycle. Next, samples were transferred to new 1.5 ml Eppendorf tubes and centrifuged for 10 min at 10,000 *g* at 20°C. Protein amounts were determined using a Pierce™ Rapid Gold BCA Protein Assay Kit (Thermo Fisher Scientific).

Aliquots (50 μg) of each protein suspension were next reduced during 20 min using a 10 mM final concentration of dithiothreitol and alkylated with a 20 mM final concentration of iodoacetamide for 20 min in the dark, both at 37°C. Subsequently, sample buffer was immediately removed using the Single-Pot Solid-Phase-enhanced Sample Preparation (SP3) as described previously (Müller et al., 2020). Briefly, 500 μg of Sera-Mag SpeedBeads Hydrophilic (GE Healthcare) were added to samples (10/1 w/w beads-to-protein ratio), then acetonitrile (ACN) was added to a final concentration of 75%. The beads were agitated with a thermoshaker for 10 min at 500 rpm at room temperature followed by 2 min of incubation in a magnetic rack. The supernatant of each sample was removed, and beads were washed twice with 200 μl of 80% ethanol and once with 200 μl of neat ACN. Beads were resuspended in 90 μl of 100 mM ammonium bicarbonate solution containing 1 μg of Trypsin/Lys-C Protease Mix (1/50 w/w enzyme-to-protein ratio; Thermo Fisher Scientific). Enzymatic digestion was carried out for 2 h on a thermoshaker at 37°C with 500 rpm agitation. Following digestion, the samples were sonicated for 5 min in a sonic bath, centrifuged for 1 min, and incubated for 2 min on the magnetic rack, then 90 μl was transferred to a new plate and 10 μl of 20% ACN / 5% trifluoroacetic acid was added to quench enzymatic digestion.

Reverse phase separation of 1 μg of digest was done on an Ultimate 3000 RSLC Nano system using a 25 cm column (75 μm internal diameter, Reprosil-Pur C18-AQ 1.9 μm phase, PepSep) at a flow rate of 500 nl.min^−1^ and maintained at 60°C. Solvent A was 0.1% formic acid in LC–MS grade water and solvent B was 0.1% formic acid in 80% ACN. Gradient consisted of a first increase of solvent B from 10 to 30% over 83 min then from 30 to 55% over 17 min. Total run time including column wash and re-equilibration was 120 min.

Tandem mass spectrometry analysis was performed on a Q Exactive mass spectrometer (Thermo Fisher Scientific) equipped with an Easy-Spray nanosource. A full scan was acquired between 350 and 1,400 *m/z* (mass to charge ratio) at a resolution of 70,000 [automatic gain control (AGC) target of 3.10^6^ ions or 50 ms maximal injection time]. The top 20 precursors were then selected for MS2 analysis at a resolution of 17,500 [AGC target of 1.10^5^ ions, 50 ms maximal injection time, and normalized collision energy (NCE) 27%] with an isolation window of 2 *m/z*.

Extraction and annotation of MS spectra were performed using Proteowizard (v. 3.0.9992; Chambers et al., 2012), OpenMS (v. 2.4; Röst et al., 2016) software, and BIOTRACS, an in-house solutions developed with MATLAB (R2019). Raw MS spectra were processed using peak picking algorithm to annotate most significant peaks using their *m/z* ratio and retention time (RT). All peaks below a predetermined background-intensity threshold of 1.10^4^ were removed from data. This threshold was defined according to the mass spectrometer characteristics. The retention time of remaining features were next aligned across all samples to correct potential RT drifts during the chromatography. Peptides were identified by using MASCOT search engine against the *Bordetella pertussis* Tohama I proteome (proteome ID UP000002676) and regrouped in protein by Fido algorithm (Serang et al., 2010). Trypsin was specified as the enzyme, cleaving after all lysine and arginine residues and allowing up to two missed cleavages. Carbamidomethylation of cysteine (+ 57.021 Da) was specified as fixed modification and oxidation of methionine (+ 15.995 Da) was considered as variable modification. A false discovery rate of less than 5% was applied to both peptide spectral matches and protein based on linear discriminant analysis using a target decoy strategy (Elias and Gygi, 2007).

A filter was applied to keep the features present in more than 80% of samples per group. Subsequently, using BIOTRACS, several QC analyses were performed on processed data mainly to validate homogeneity between samples, considering proteins identified per sample, peptides miss cleavages, samples intensity, and proportion of peptides number used for protein identification. Protein intensities were converted in Log_2_ scale and normalized by the subtraction of the median intensity of each sample (Dubois et al., 2022).

### Lipidomic data generation and analysis

Lipidomics is defined as the full characterization of lipid molecular species and of their biological roles (Raghunandanan et al., 2019; Bale et al., 2021). Lipids were extracted using Bligh and Dyer liquid extraction method (Bligh and Dyer, 2011; Sündermann et al., 2016). Briefly, 700 μl of the mix chloroform (CHCl_3_)/methanol (MeOH) at the ratio 1/2 (v/v) were added to pellets. Samples were homogenized by pipetting, transferred in 2 ml vials containing the lysing matrix B (MP Biomedicals) and lysed by bead beating using the Precellys Evolution homogenizer (Bertin Technologies; 3 × 40 s, 6,500 rpm, 4°C, pause of 30 s). Then, samples were incubated in a thermoshaker (Eppendorf) for 10 min at 1,600 rpm and 4°C and centrifuged for 3 min at 10,000 *g* and 4°C. For each sample, 500 μl of supernatants were isolated. The extraction was repeated two more times with 700 μl of the mix CHCl_3_/MeOH/H_2_O at the ratio 1/2/0.8 (v/v), and 2 × 700 μl of supernatants were isolated. Aqueous and organic phases were separated by addition of 500 μl of H_2_O and CHCl_3_, mixed by vortexing and then centrifuged for 5 min at 2,500 g at room temperature to accelerate phase separation. Organic phase was isolated (1,000 μl), and 800 μl of CHCl_3_ was added to repeat organic extraction. Samples were mixed, centrifuged, and organic phase was isolated (800 μl). Then, solvents were evaporated under nitrogen flow at 40°C using the Stuart®evaporator for 1 h. Dried samples were stored at −20°C waiting analysis. As a control, blank samples without biological materials were also prepared using the same preparation protocol.

Lipids were dissolved in 200 μl of isopropanol (IPA) containing an internal standard (IS) and incubated for 10 min at 1,600 rpm and 10°C. This IS is composed of 1 μg.ml^−1^ of lyso-phosphatidylethanolamine (lyso-PE; 17,0), arachidonic acid (C20:4 D8), PE (C16:0 D31/18:1), phosphatidylglycerol (PG; C16:0 D31/18:1), phosphatidylcholine (PC; C14:0 /14:0 D54), and Ceramide (16,0 D31) in IPA. Then, 25 μl of each sample were pooled to obtain QC samples and the rest of the samples was transferred in HPLC vial inserts for LC–MS analysis. IPA and IPA with IS were used as blanks for LC–MS analysis.

Intracellular lipids were analyzed using an Ultra-High Performance Liquid Chromatography-High-Resolution Mass Spectrometry (UHPLC-HRMS) and a Thermo Scientific Vanquish UHPLC System coupled to a Q Exactive HF mass spectrometer. Three separate injections were performed to acquire spectra in positive and negative ionization modes. Data were acquired in full scan alternating with data dependent acquisition (top 10) to obtain MS/MS spectra.

Lipids were separated on a C18 CSH column (100 mm × 2.1 mm, 1.7 μm, Waters) at 55°C (Isaac et al., 2011). Flow rate was 400 μl.min^−1^. The mobile phase A consists of ACN/H_2_O (60/40, v/v) with 10 mM ammonium formate, and 0.1% formic acid and mobile phase B IPA/ACN (90/10, v/v) with 10 mM ammonium formate and 0.1% formic acid. The injection volume was 5 μl. The gradient used for the lipid profiling by UHPLC–MS was performed according to previous study (Isaac et al., 2011).

Q Exactive HF mass spectrometer (Thermo Fisher Scientific) is equipped with HESI-II probe. A full scan was implemented on both positive and negative mode with mass range between 150 and 1,250 *m/z* (Isaac et al., 2011). The ion source settings were as follows: spray voltage = 3.5 kV for electrospray ionization (ESI) in positive mode and 2.7 kV for negative ESI, capillary temperature = 320°C, sheath gas flow rate = 40, and auxiliary gas = 10 and sweep gas = 0. Data acquisition settings were with AGC (MS1) = 3.10^6^ ions, mass range = 150–2,000 *m/z*, injection time = 100 ms, and resolution = 120,000. Data acquisition for fragmented sequences was also applied on separated injections, experimental settings were AGC (MS2) = 1.10^5^ ions, mass range = 150–2,000 *m/z*, injection time = 100 ms, and resolution = 30,000, Top 10, dynamic exclusion = 10 s and NCE = 20–30—40%.

Extraction and annotation of MS spectra were performed using Proteowizard (v. 3.0.9992; Chambers et al., 2012) and OpenMS (v. 2.4; Röst et al., 2016) software, and BIOTRACS. Raw MS spectra were processed using peak picking algorithm to annotate most significant peaks using their *m/z* ratio and RT. All peaks below a predetermined background-intensity threshold of 1.10^4^ were excluded from data. This threshold was defined according to the mass spectrometer characteristics. The retention times of remaining features were next aligned across all samples to correct potential RT drifts during the chromatography. The resulting feature table was used for QC analysis to correct analytical drifts, in particular intra-batch effects. The coefficient of variation (CV = standard deviation/mean) of features was computed to assess the analytical stability of the features. Features with a CV greater than 30% in QC samples were removed from the data because they were considered as analytically inconsistent. A filter was applied to keep the features present in 80% in the QC samples. Positive and negative mode data were next merged.

Metabolic features were identified using LipidMatch R software (Koelmel et al., 2017). In LipidMatch, a ranking was done on the detected metabolites: Level 1: precursor mass found and confirmed by MS/MS fragments; Level 2: does not apply to our experiment as it refers to all ion fragmentation; Level 3: found class of lipids; Level 4: found precursor mass but unfound MS/MS fragments; and Level 5: unfound precursor mass in the database of LipidMatch. Lipid intensities were converted in Log_2_ scale and normalized by the subtraction of the median intensity of each sample.

### Extracellular metabolomic data generation and analysis

Supernatant thawed at room temperature were vortexed and 250 μl of samples were filtered using 0.2 μm centrifugal filter units (VWR; for 5 min at 10,000 *g* and 4°C). The supernatants were additionally filtered using 10 kDa Molecular weight cut-off (MWCO) centrifugal filter units (VWR; for 15 min at 10,000 *g* and 4°C). The 45 μl of resulting filtrates were mixed with 90 μl of water and 45 μl of Sodium trimethylsilylpropanesulfonate (DSS) internal standard solution at pH 6, containing phosphate buffer and D_2_O for the signal lock. The resulting concentration of the DSS was about 0.5 mM. The obtained solutions were vortexed and 155 μl were transferred in 3 mm SampleJet NMR tubes (Bruker®). Finally, tubes were loaded in the 4–6°C pre-cooled SampleJet autosampler before analysis. In addition to samples, blank samples were also prepared using the same preparation protocol, with MilliQ water instead of culture supernatants. The DSS concentration was calibrated with 1.305 mM sodium succinate dibasic hexahydrate solution in triplicates in order to guarantee data accuracy.

The sample analysis was performed using Ascend 600 MHz (Avance III HD) NMR spectrometer (Bruker Biospin) equipped with cryogenically cooled 5 mm QCI (^1^H/^13^C/^15^N/^31^P) probe head. For each sample a one-dimensional proton acquisition was performed using *noesygppr1d* pulse sequence, which contain a pre-saturation block for water attenuation during relaxation delay. The spectral width of proton acquisition was 14 ppm using 64 k data points during 3.8 s acquisition time and 4 s relaxation delay. The mixing time delay was 70 ms. Spectra for extracellular metabolites contained 512 scans for about 70 min of total acquisition time. The free induction decay (FID) raw data were processed by Fourier transformation using 0.3 Hz exponential apodization function within 64 k data points. The resulting spectra were phase and baseline corrected. The spectra alignment was done according to internal standard DSS chemical shift. Finally, the metabolites quantification was performed using Chenomx NMR suite 8.3. The metabolites concentrations were reported in mM and exported in Excel format file for statistical analysis.

### Biostatistics analyses

Each omics dataset was processed independently through a unique statistical pipeline consisting of three steps: QCs, supervised univariate analysis, and longitudinal clustering. An additional pathway enrichment step was also performed on transcriptomic and proteomic data.

For QCs, principal component analyses (PCA) and hierarchical clustering (based on Ward distance) were used to measure biological variability and ensure samples were more similar within (technical and biological replicates) than across time points.

A variance partition step was also conducted to investigate the relative contribution of time, technical variables (sequencing batch, bioreactor), and noise on omics measurements. This step was carried out using the Gaussian Processes R package *lgpr* (Timonen et al., 2021).

Univariate analysis was done using both (moderated) *t*-test and Gaussian Processes to quantify the effect of time on expression data and measure differences between time points. A linear mixed model was fitted on transcriptomic and proteomic data using Limma (combined with Voom in the former case; Law et al., 2014) to account for the correlation between samples from the same bioreactor. The time and batch effects were, on the other hand, included in the model as categorical fixed effects. For each gene/protein, a moderated *t*- and *F*-statistics were computed to test whether pairwise differences among time points (contrasts) were equal to zero separately and altogether. *F*-statistics were used as a measure of significance of the time effect along the whole trajectory. To identify major variations, the significativity threshold for gene/protein variations was fixed to |log_2_FC| > 1 with an adjusted *p*-value < 0.05. To identify fine changes of specific genes transcription or proteins syntheses, variations with a |log_2_FC| > 0.5 and an adjusted *p*-value < 0.05 were scrutinized. For other omics than transcriptomic and proteomic, a paired *t*-test was used in place of Limma.

Unlike linear mixed model, Gaussian processes are non-parametric approaches that model more complex covariance structures and detect non-linear effects of both categorical and continuous covariates (as well as their interactions). The *lgpr* package provides a measure of covariate effects (proportion of explained variance) for each omics feature as well as a measure of covariate relevance. Using the same three effects (time, sequencing batch and bioreactor), we found that the percentage of explained variance returned by *lgpr* led to an almost identical gene ranking as linear mixed model *F*-statistics.

A longitudinal clustering was then carried out using Dirichlet process Gaussian process mixture model (DPGP; McDowell et al., 2018) that combines a Dirichlet process to determine the number of clusters and a Gaussian process to model the trajectory along the time. Both approaches are based on nonparametric methods. For each omic, only the features with an explained variance larger than 25% were fed into the model, threshold set to filter out non-informative features. The 25% value was found to correspond to the 5% adjusted *p*-value in the Limma-lgpr comparison on transcriptomic data. DPGP returned clusters of omics features presenting shared trajectories along the time.

A last step of pathway enrichment was performed on transcript and protein clusters, assuming that features with similar trajectories shared biological functions. A standard over-representation analysis based on the Fisher’s exact test (Huang et al., 2009) was carried out on Kyoto Encyclopedia of Genes and Genomes (KEGG) and Gene Ontology (GO) databases using clusterProfiler R package (Yu et al., 2012).

## Results

### Standardized growth and biosynthesis of vaccine antigens through experimental cultures

**Figure 2:**
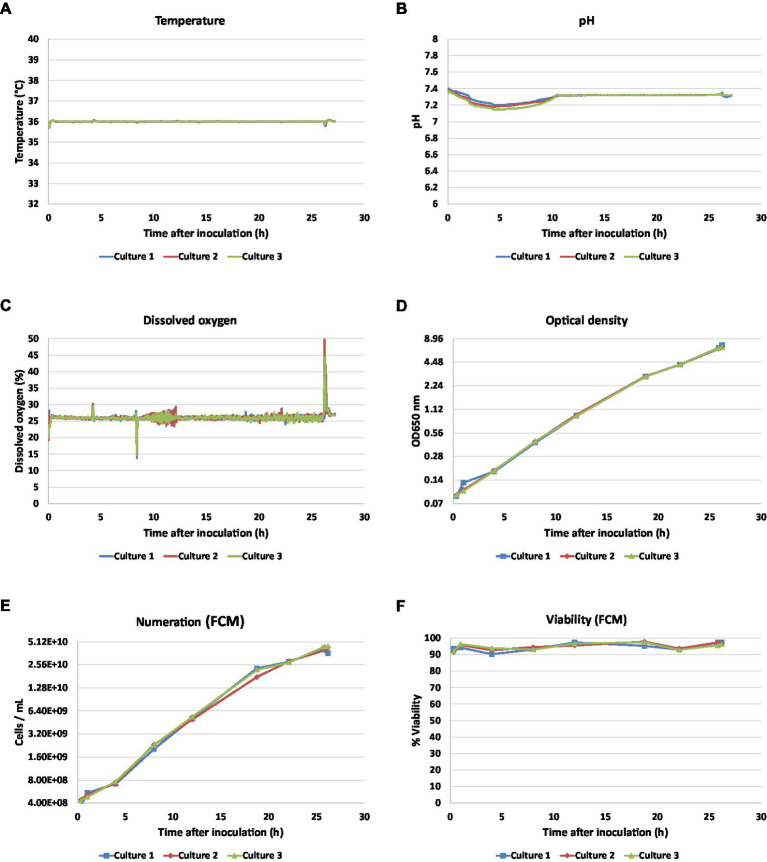
Monitoring profiles of three independent bioreactor cultures of *Bordetella pertussis* Tohama I. Temperature **(A)**, pH **(B)**, DO **(C)** were monitored inline by Ambr system, OD_650_ **(D)**, bacteria numeration by flow cytometry **(E)**, and viability measured by flow cytometry **(F)** were offline determined on 8 time points.

To mimic industrial culture conditions, three independent culture batches were performed in hydrolysate casein-like (HC-like) chemically defined medium (Izac et al., 2015). As expected, no significance difference in temperature, pH, and dissolved oxygen (DO) were observed throughout cultures (Figures 2A–C). Similar and minor perturbations were observed regarding pH regulation at the beginning of the culture and dissolved oxygen fluctuation linked to sampling in the three fermenters. Bacterial growth and viability were monitored by OD_650_ measurements and flow cytometry analyses (Figures 2D–F). Independent culture replicates showed similar growth and a viability higher than 90% was observed throughout cultures. The end of culture was identified by a DO peak and a CER drop at 26 h. Two main growth phases could be distinguished during culture: the first, between 0 h and 18 h 45 min (early to middle logarithmic phase), was characterized by an OD and a numeration growth rate of 0.19 and 0.21 h^−1^ respectively, the second, between 18 h 45 min and 26 h (mid to late log phase), differed by lower growth rate (0.12 and 0.09 h^−1^, for OD and numeration respectively).

Final amounts of PT, PRN, Fim2, and FHA were measured from whole bacterial suspensions using ELISA. The mean antigen concentrations were 10.5 μg.ml^−1^ (CV = 3.0%) for PT, 24.2 μg.ml^- 1^ (CV = 4.6%) for PRN, 211.6 ng.ml^−1^ (CV = 2.7%) for Fim2, and 150.4 μg.ml^−1^ (CV = 1.8%) for FHA. Based on the small coefficient of variations observed across batches, we thus extrapolated that our experimental biological triplicates were similar.

### Longitudinal multi-omics reveals a global metabolic switch during *Bordetella pertussis* culture

**Figure 3:**
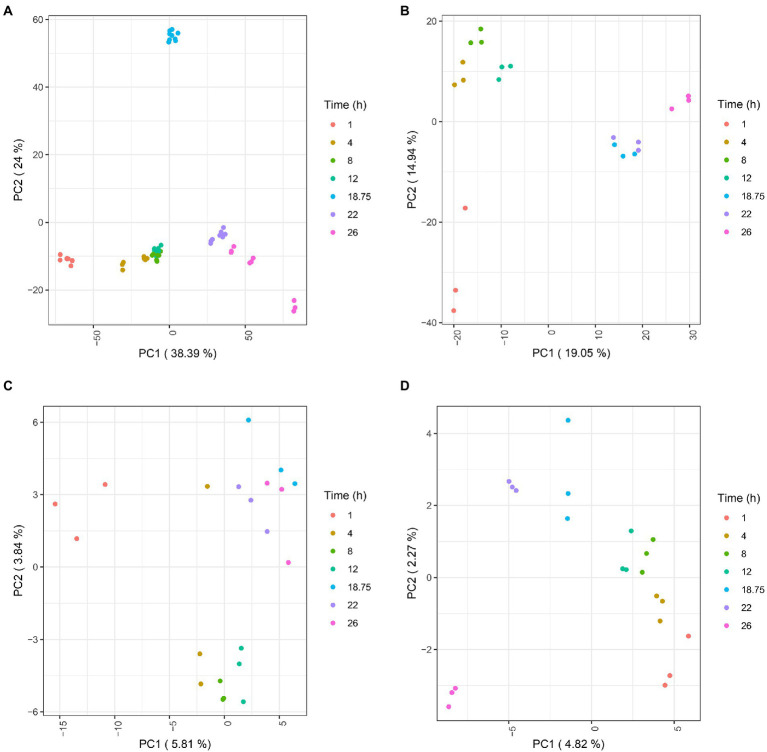
PCA plots of transcriptomic **(A)**, proteomic **(B)**, lipidomic **(C)**, and extracellular metabolomic **(D)** data. For transcriptomic, each single dot represents a library replicate. For other omics, each single dot represents a biological replicate.

Transcripts, proteins, lipids, and metabolites were analyzed from three independent batch cultures at different times points (T = 1 h, 4 h, 8 h, 12 h, 18 h 45 min, 22 h and 26 h; Figure 1). Following raw data processing, a total of 3,581 transcripts, 1,340 proteins, 99 lipids, and 37 extracellular metabolites were identified and quantified (Supplementary Tables 1–4). PCA was conducted on each omics dataset separately to visually check the consistency between the biological replicates and to evaluate the relative contributions of experimental factors on data variation (Figure 3). PCA within each omics revealed that samples were clustered according to time points, reflecting a high homogeneity across biological replicates and therefore, high reproducibility in all omics. As expected from the literature (Metz et al., 2017; Dienstbier et al., 2019), intra time point variation was higher in proteomic and lipidomic data due to the larger technical variability and weaker variation of lipid and protein amounts during culture compared to transcripts. Due to technical sampling issues that were later confirmed by the PCA analysis, proteomic and lipidomic samples collected at T = 1 h were excluded from downstream analyses. After data quality assessment, statistical longitudinal analyses were performed.

We first aimed at identifying the feature variations that were the most associated with time using Longitudinal Gaussian Processes (LGPR; Timonen et al., 2021; Supplementary Tables 1–4). Gaussian processes are flexible non-parametric approaches, able to model complex non-linear and non-stationary signals. In agreement with initial PCA, proteomic and lipidomic data showed higher technical variations than transcriptomic and extracellular metabolomic data. Features with more than 25% of their variance explained by the time were considered of interest in downstream analyses. This threshold was found to correspond approximately to a 5% *q*-value in a linear mixed model (see Material and Methods).

Overall, 3,080 out of 3,581 transcripts, 723 out of 1,340 proteins, 42 out of 99 lipids, and 33 out of 37 extracellular metabolites passed this cut-off.

**Figure 4:**
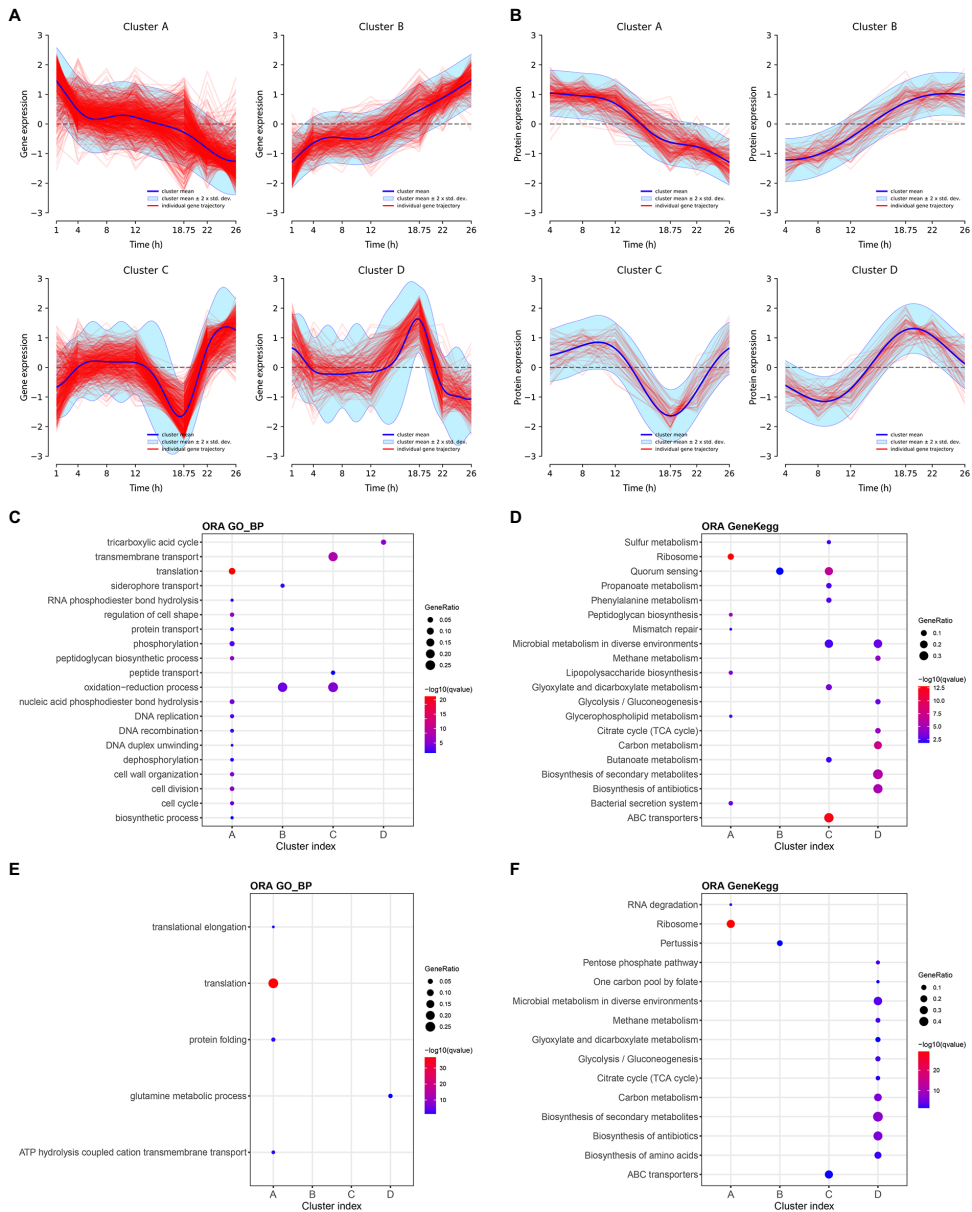
Main longitudinal clusters trajectories and pathway enrichment results of transcriptomic and proteomic data of *Bordetella pertussis* culture. Main longitudinal clusters trajectories of transcriptomic **(A)** and proteomic **(B)** data. *X*-axis correspond to the time and *Y*-axis correspond to the gene expression or the protein intensity. Blue line corresponds to the cluster mean, light blue interval corresponds to the cluster mean ± 2 × SD and red line corresponds to the individual transcript or protein trajectories. Over representation analysis (ORA) pathways enrichment on Gene Ontology and KEGG databases for transcriptomic (**C,D**, respectively) and proteomic (**E,F**, respectively). Enriched pathways are shown in *Y*-axis and associated cluster in *X*-axis. Colors of spots (blue to red) are according to the significance of pathways enrichment in each cluster. Size of spots are according to the Gene ratio (= number of transcripts or proteins in a cluster which mapped on a pathway/Number of transcripts or protein in this cluster which mapped in the database). Maximum number of pathways = 20. Maximum *q*-value = 0.05.

Using the features selected above, we subsequently investigated similarities among feature trajectories using longitudinal clustering in each omics dataset (McDowell et al., 2018). Cluster analyses identified 13 and 11 clusters in transcriptomic and proteomic data, respectively (Supplementary Figures 1, 2). Next, clusters with similar profiles were merged and regrouped in larger clusters named A–D for both transcriptomic and proteomic data (Supplementary Tables 1, 2). For the ease of graphic representation, representative profiles of these large clusters are depicted in Figures 4A,B.

All clusters trajectories are globally monotonous up to 12 h of culture. After 12 h, the profiles of clusters A (1,132 transcripts and 260 proteins) decrease while the profiles of clusters B (657 transcripts and 81 proteins) increase until the end of culture. Clusters C are composed of 471 transcripts and 88 proteins and decreases over 12 h, before going up after 18 h 45 min until the end of culture. Conversely, the profile of clusters D (391 transcripts and 130 proteins) increases after 12 h, up to 18 h 45 min before decreasing until the end of culture. The consistency between proteins and transcripts clusters was evaluated by measuring the percentage of proteins from a cluster profile found within their matching transcripts in the same cluster profile. The consistency between proteins and transcripts was 70, 51, 65, and 52%; for the clusters A–D, respectively. Moreover, between 12 h and 18 h 45 min, 1,790 genes (177 up- and 309 down-regulated, |log_2_FC| > 1) and 399 proteins (17 up-, including five ON proteins, and 17 down-regulated, including eight OFF proteins, |log_2_FC | > 1) are differentially expressed (adj *p*-value < 0.05; Supplementary Tables 5, 6). Between 18 h 45 min and 22 h, 1,671 genes (310 up and 91 down regulated) and 90 proteins (five up including three ON proteins and zero down regulated) were differentially expressed (adj value of *p* < 0.05). Finally, clustering reveals a specific event at 18 h 45 min of culture with irreversible impact highlighted by both clusters A and B. Both clusters C and D indicate a steep change of omics profiles before returning to their respective general paths of expression suggesting a transient physiological state.

To functionally characterize clusters, pathway enrichment analyses (Huang et al., 2009) were next performed with the groups of transcripts/proteins that constitute each cluster using the KEGG and GO databases (Supplementary Tables 7–10). For each database, the top 20 pathways (ranked by *q* value, *q* values <0.05) identified with the transcriptomic and proteomic data of the clusters are, respectively, reported in the Figures 4C,D. The cluster A, both in transcriptomic and proteomic, is mainly associated with several biosynthesis pathways, such as ribosome, translation, peptidoglycan biosynthesis, and lipopolysaccharide biosynthesis pathways. Over time, after 12 h of culture, pathways of the cluster A were less used by *B*. *pertussis*. This finding is consistent with the observation of a growth rate reduction. The decrease of such biosynthesis pathways was previously highlighted during the transition from exponential to stationary phase as well as under glutamate limitation (Nakamura et al., 2006). Cluster C enrichment revealed that the expression of 55 ABC transporter genes and the synthesis of 15 ABC transporter proteins decrease specifically at 18 h 45 min of culture. Cluster D was enriched with transcripts and proteins involved in TCA cycle and glycolysis/gluconeogenesis pathways.

The decrease of multiple biosynthesis pathways and the increase of ABC transporter genes after 18 h 45 min point toward one or several putative culture medium components starvation.

**Figure 5:**
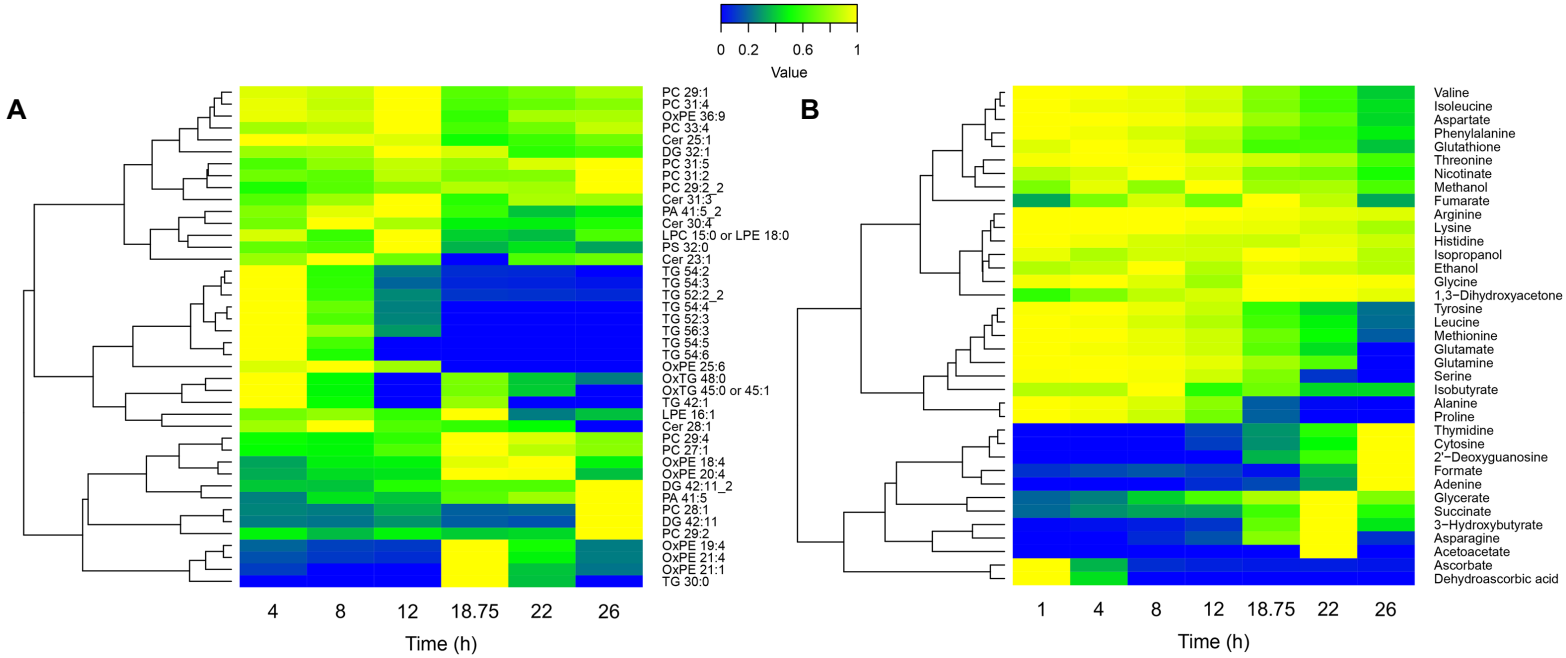
Lipidomic **(A)** and extracellular metabolomic **(B)** longitudinal data heatmaps of *B*. *pertussis* culture. Lipid intensities and metabolite concentrations are represented from 0 (blue) to 1 (yellow), 1 representing the maximum intensity/concentration of every feature. Features are clustered according to their trajectory similarities throughout the culture. Cer, ceramide; DG, diglycerides; LPC, lysophosphatidylcholine; LPE, lysophosphatidylethanolamine; Ox, oxydated; PA, phosphatidic acid; PC, phosphatidylcholine; PE, phosphatidylethanolamine; PG, phosphatidylglycerol; PS, phosphatidylserine; and TG, triglycerides.

To confirm this hypothesis and further decipher the physiology behind the breakpoint occurring at 18 h 45 min, the longitudinal lipidome and extracellular metabolome of Tohama I were investigated (Figures 5A,B). The lipidomic analyses showed different trends of lipid variation across the lipidome (Figure 5A). Phosphatidylcholine (PC) mostly accumulated at the end of the culture. Oxydated phosphatidylethanolamine (OxPE) increased at 18 h 45 min, which suggests an occurring oxidative stress. Conversely, triglycerides (TG) were totally degraded at 18 h 45 min. Extracellular metabolomics analyses showed large variations of metabolite concentrations in the culture medium along the culture process (Figure 5B). In agreement with previous studies performed in modified SS-medium, purine (adenine), pyrimidine (cytosine) and nucleosides (deoxyguanosine and thymidine) accumulated at the end of the bioreactor culture (Branco dos Santos et al., 2017). In addition, several other metabolites, such as acetoacetate, hydroxybutyrate, and succinate also accumulated from 18 h 45 min to 22 h.

Diverse carbon sources consumption could be noticed. Among those carbon sources, alanine and proline were completely consumed between 18 h 45 min and 22 h (Figure 5B). Proline is known to be one of the main carbon sources in modified SS-medium (Stainer and Scholte, 1970; Izac et al., 2015) with glutamate. Thereby, we hypothesized that the observed growth rate decrease, the biosynthesis pathways slowdown, the energy storage consumption, and the marked modulation at 18 h 45 min revealed by longitudinal clustering analysis may be the consequences of a proline starvation. To validate this hypothesis, proline and glutamate metabolisms were next investigated.

### Proline starvation induces transient metabolism with energy stock consumption and impacts central metabolism

**Figure 6:**
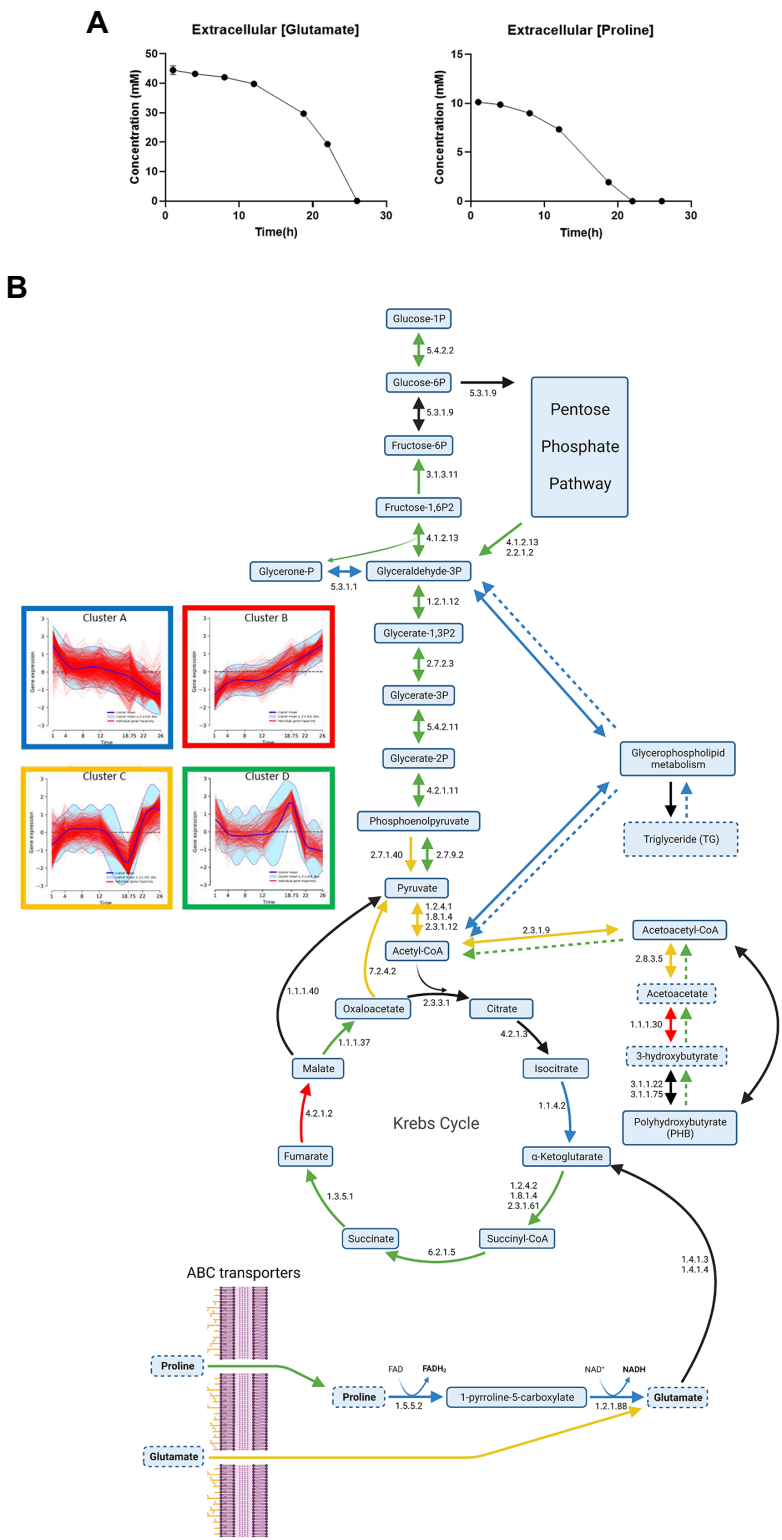
Impact of proline starvation on central metabolism during *Bordetella pertussis* Tohama I culture. **(A)** Longitudinal evolution of glutamate and proline extracellular concentrations monitored by NMR analyses. **(B)** Central metabolism gene expression profiles during *B*. *pertussis* Tohama I culture. Blue, red, yellow, and green arrows show the enzymes encoded by genes of the clusters A–D (Figure 4A), respectively. Black arrows indicate a discrepancy of cluster between the different enzyme genes. Numbers next to arrow reactions are the EC numbers of the reaction. Dashed arrows are reactions deducted from lipidomic or metabolomic results using the same color code than transcriptomics data. Lipids/Metabolites with dashed outlines were quantified in lipidomic or extracellular metabolomic analyses. This figure was created with BioRender.com.

The concentrations of glutamate and proline were quantified in the culture medium along the culture (Figure 6A). Extracellular sources of proline and glutamate were fully consumed after 22 and 26 h of culture, respectively. Thus, we can assume that a proline starvation occurs between 18 h 45 min and 22 h, while 44% of the initial extracellular glutamate is still available after 22 h of culture. Therefore, we hypothesized that a decrease of proline catabolism followed by a compensatory increase of glutamate uptake should simultaneously occur between 18 h 45 min and 22 h of culture. To verify this hypothesis, proline and glutamate metabolisms were investigated through an analysis of transcriptomic (Figure 6B) and proteomic data.

On one hand, proline ABC transporter genes (*bp2055*, *bp2056*, and *bp2057*) expression decreased (log_2_FC = −1.5) between 1 and 4 h. The expression of these genes remained stable between 4 and 12 h, increased from 12 h to 18 h 45 min, and then decreased from 18 h 45 min to 26 h of culture (Kyoto Encyclopedia of Genes and Genomes, 2022; Supplementary Table 1). Following a 22 h preculture, *B*. *pertussis* bacteria have consumed the stock of proline available from the preculture medium and are on starvation. The high expression of proline transporter genes at 1 h could thus be explained by the exposure to the high proline concentration of the fresh culture medium which triggers the expression of proline uptake genes.

Following its uptake, proline is known to be metabolized in glutamate using two enzymatic reactions (EC 1.5.5.2 and 1.2.1.88), producing 1 FADH_2_ and 1 NADH, mainly catalyzed by PutA (proline dehydrogenase, BP2749; Surber and Maloy, 1998). Then glutamate may be converted into 2-oxoglutarate and joins TCA cycle and central metabolism (Figure 6B). This is consistent with the sudden decrease of the *putA* gene (cluster A) expression (Log_2_FC = −4.7) and of the PutA protein (cluster A) synthesis (Log_2_FC = −1) between 12 and 22 h (Supplementary Tables 5, 6). Taken together, these results indicate that, proline uptake decreased after 18 h 45 min and the activity of the main proline catabolism pathway decreased after 12 h, in agreement with the proline starvation observed between 18 h 45 min and 22 h.

On the other hand, glutamate ABC transporter genes (*gltJ* = *bp0057*, *gltK* = *bp0055/BP0767*, *gktJ* = *bp0056*, and *gltL* = *bp0768*) expression levels were globally stable until 18 h 45 min, and then increased until the end of the culture (cluster C; Kyoto Encyclopedia of Genes and Genomes, 2022). The expression levels of other glutamate transporter genes such as gltS, encoding a sodium/glutamate symporter, increased between 12 and 22 h (log2FC = +0.8). Similarly, while the expression of *bp3831*, encoding a putative glutamate transporter (Keidel et al., 2018) decreased between 12 h and 18 h 45 min (Log2FC = −1.8), a marked expression increase of this gene was also observed between 18 h 45 min and 22 h (Log2FC = +2.0), (Supplementary Tables 5, 6). Those results show that transcriptomic and proteomic data are consistent with extracellular metabolomic data and confirm that glutamate uptake increases after proline starvation.

Proline metabolization is a major source of energy. Thus, a starvation of this amino acid conducts to a loss of energy which may trigger energy internal stock consumption. Proline is metabolized by TCA cycle *via* glutamate and poly-β-hydroxybutyrate (PHB) is connected to central metabolism *via* acetyl-CoenzymeA (acetyl-CoA). Thereby, the expression of genes and the synthesis of proteins involved in the central metabolism were investigated (Figure 6B). In *B*. *pertussis*, the TCA cycle was recently demonstrated to be entirely functional in the Tohama I strain (Izac et al., 2015; Branco dos Santos et al., 2017; Gonyar et al., 2019). In the present study, the expression of most TCA cycle genes and the synthesis of corresponding proteins increased at 18 h 45 min of culture (cluster D; Figure 6B; Supplementary Tables 1, 2). Transcripts and proteins involved in the conversion of citrate to isocitrate and 2-oxoglutarate (EC 4.2.1.3 and 1.1.1.42) decreased after 12 h of culture. Similarly to a large part of the TCA cycle, determinants of gluconeogenesis activity increased at 18 h 45 min of culture (cluster D). In *B*. *pertussis*, gluconeogenesis is necessary to supply carbon to several pathways including the pentose phosphate pathway (PPP) and the glycerophospholipid metabolism (Figure 6B). PPP is used to synthetized DNA precursors (Kyoto Encyclopedia of Genes and Genomes, 2022). Thus, the reduced growth and the decrease of several biosynthetic activities are consistent with the slowdown of central metabolism after 18 h 45 min of culture. The expression of most of glycerophospholipid metabolism genes decreased at 18 h 45 min (cluster A). As glycerophospholipid metabolism allows to synthetize triglycerides, this observation is in agreement with the absence of triglyceride at 18 h 45 min (Kyoto Encyclopedia of Genes and Genomes, 2022).

Taken together, these findings strongly suggest a proline starvation at 18 h 45 min of culture in modified SS-medium leading to a major modulation on *B*. *pertussis* central metabolism. As modified SS-medium is a commonly used medium for *B*. *pertussis* cultivation (Fyson et al., 2017), in laboratory and industrial processes, the impact of this metabolic switch was further assessed on antigen production and virulence.

### *Bordetella pertussis* antigen production and global virulence regulation during culture

**Figure 7:**
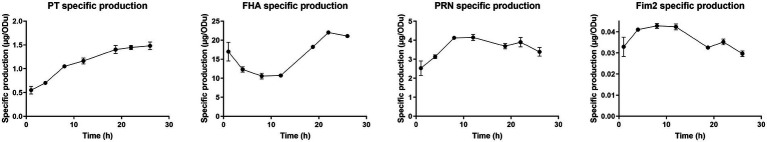
Specific antigen production throughout *Bordetella pertussis* culture. ELISA antigen quantification was performed for each antigen on lysed crude harvest and normalized using biomass measured with OD_650_.

Antigens used in aP vaccines are *B*. *pertussis* virulence factors. To document major antigens production and evaluate the impact of proline starvation on their production, a longitudinal antigen quantification was carried out (Figure 7).

The specific total production of PT increased by 3-fold (0.55–1.48 μg.ODu^−1^) from the beginning to the end of culture, with a marked slowdown of after 18 h 45 min. Indeed, specific production speed exhibits non-significant 3-fold decrease after 18 h 45 min compared to the 12–18 h 45 min interval. The specific total FHA production was stable between 4 and 12 h and then sharply increased until 22 h by 2-fold (10.73–22.00 μg.ODu^−1^). The specific total PRN and Fim2 production increased from 1 to 8 h after inoculation, remained stable from 8 to 12 h and then slightly decreased until the end of culture (between 12 and 26 h).

Transcriptomic and proteomic analyses were used to monitor transcripts and proteins involved in aP vaccine antigen production throughout culture (Supplementary Tables 1, 2, 5, 6). Data showed that *ptx* operon transcription increased throughout culture (log_2_FC ≈ + 1 between the beginning and the end of culture) and Ptx subunits amounts slightly increased until 18 h 45 min of culture (log_2_FC ≈ +0.5 between 4 h and 18 h 45 min) and then remained stable (Supplementary Figure 3). PT is folded by DsbAC in the periplasm and externalized by Ptl type IV secretion system (Stenson and Weiss, 2002; Stenson et al., 2003). The transcription of *dsbA* increased from 4 h to 18 h 45 min (log_2_FC = +1.2), then decreased until 22 h (log_2_FC = −0.6 between 18 h 45 min and 22 h) and increased until the end of the culture (log_2_FC = +0.7 between 22 and 26 h). The amount of *dsbC* transcripts and DsbA/DsbC concentrations were stable throughout the culture. *Ptl* operon expression and the corresponding protein concentrations remained stable (Supplementary Figure 4). The concentration of FhaB, the precursor of FHA, slightly varied during culture. Conversely, *fhaB* expression increased by almost two-fold (log_2_FC = +0.9) between 4 h and 18 h 45 min of culture. FhaB is transported through the outer membrane by FhaC, processed by CtpA and released by SphB1 (Nash and Cotter, 2019). Transcripts of *fhaC* increased from 8 h to 18 h 45 min (log_2_FC = +0.5) and then decreased between 18 h 45 min and 22 h (log_2_FC = +0.9). The transcriptions of *sphB1 and ctpA* were stable throughout the culture. SphB1 quantity slightly increased between 4 and 22 h (log_2_FC = +0.6) and FhaC and CtpA concentrations remained stable. *Prn* gene expression increased until 12 h and then decreased until the end of the culture (log_2_FC = −0.8 between 12 and 26 h). Similar observation was observed for PRN in proteomic but with lower variations. Fim2 antigen is composed of Fim2 and FimD subunits (Scheller and Cotter, 2015). *Fim2* gene expression was stable during the culture and *fimD* expression increased from 8 h to 18 h 45 min (log_2_FC = + 0.6) and decreased between 18 h 45 min and 22 h (log_2_FC = −0.8). Fim2 concentration was stable while FimD decreased from 18 h 45 min to 26 h (log_2_FC = −0.5). Fim2 antigens is folded by the FimB chaperone and externalized by FimC usher proteins (Scheller and Cotter, 2015). Gene expression of *fimBC* was stable from 4 h to 18 h 45 min and decreased between 18 h 45 min and 22 h (log_2_FC = −0.5-0.7). The amount of FimB was stable throughout culture while FimC concentration increased from 8 to 22 h (log_2_FC = +0.6). The transcription of *fim3* was stable throughout culture. Fim3 protein was not detected by our proteomic analysis. In agreement with ELISA data, those results suggest that *dsbA*, *fhaC*, *prn*, and *fimD* gene expressions and Ptx, Prn, and FimD protein productions might be impacted by proline starvation.

**Figure 8:**
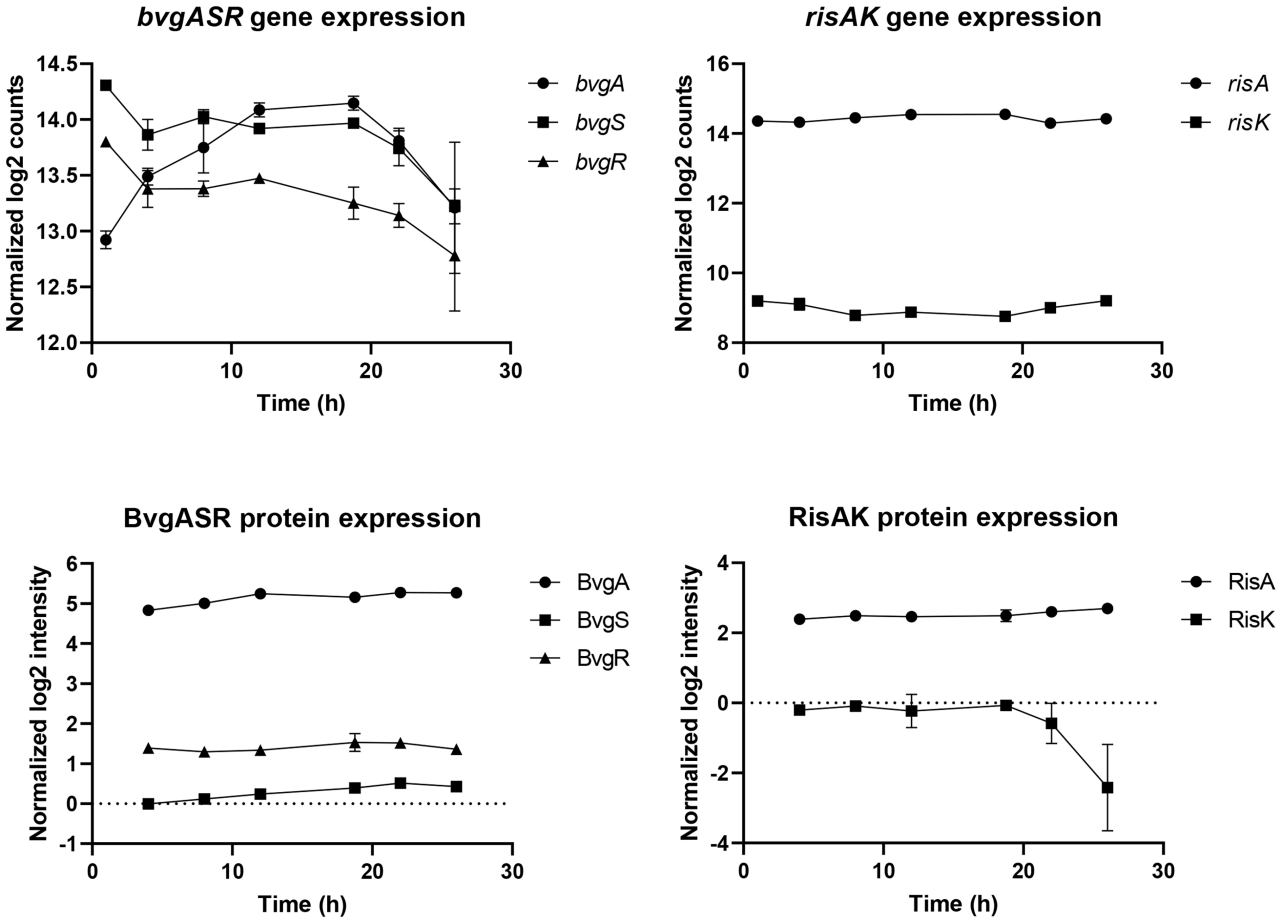
Gene and protein expression of BvgASR and RisAK regulation systems during *Bordetella pertussis* Tohama I culture.

To determine whether the proline starvation is concomitant to a global virulence modulation of *B*. *pertussis*, the master virulence regulon BvgASR profile was analyzed (Figure 8). BvgAS is a two-component system of *B*. *pertussis* that regulates the expression of virulence activated genes (*vags*). Among those genes, *bvgR* encodes for a phosphodiesterase, which turns off the expression of virulence repressed genes (*vrgs*; Chen and Stibitz, 2019). Here, *bvgA* increased from the beginning of the culture until 12 h, remained stable until 18 h 45 min, and then decreased until the end of the culture (log_2_FC = −0.8 between 18 h 45 min and 26 h). The *bvgS* gene was stable up to 18 h 45 min and then decreased until the end of the culture (log_2_FC = −0.7 between 18 h 45 min and 26 h) while *bvgR*, was stable up to 12 h and then decreased until the end of the culture (log_2_FC = −0.7 between 12 and 26 h). BvgAS protein amounts increased slightly (log_2_FC ≈ +0.5) between 4 and 22 h while BvgR remained stable. Those results suggest a moderate down regulation of *bvgASR* genes after 18 h 45 min. RisAK is a two-component system of *B*. *pertussis* involved in the regulation of virulence repressed genes (*vrgs*). In contrast to bvgASR, *risAK* transcripts and RisA proteins were stable throughout the culture. As RisK was not detected in two out of three biological replicates at 18 h 45 min, it was not possible to conclude on its variation. Finally, those data indicate that the main virulence regulation systems did not seem to be directly impacted by proline starvation at 18 h 45 min. However, the variation of their protein amounts is not informative of their activity, as they are dependent of their phosphorylation state (Luu et al., 2021).

**Figure 9:**
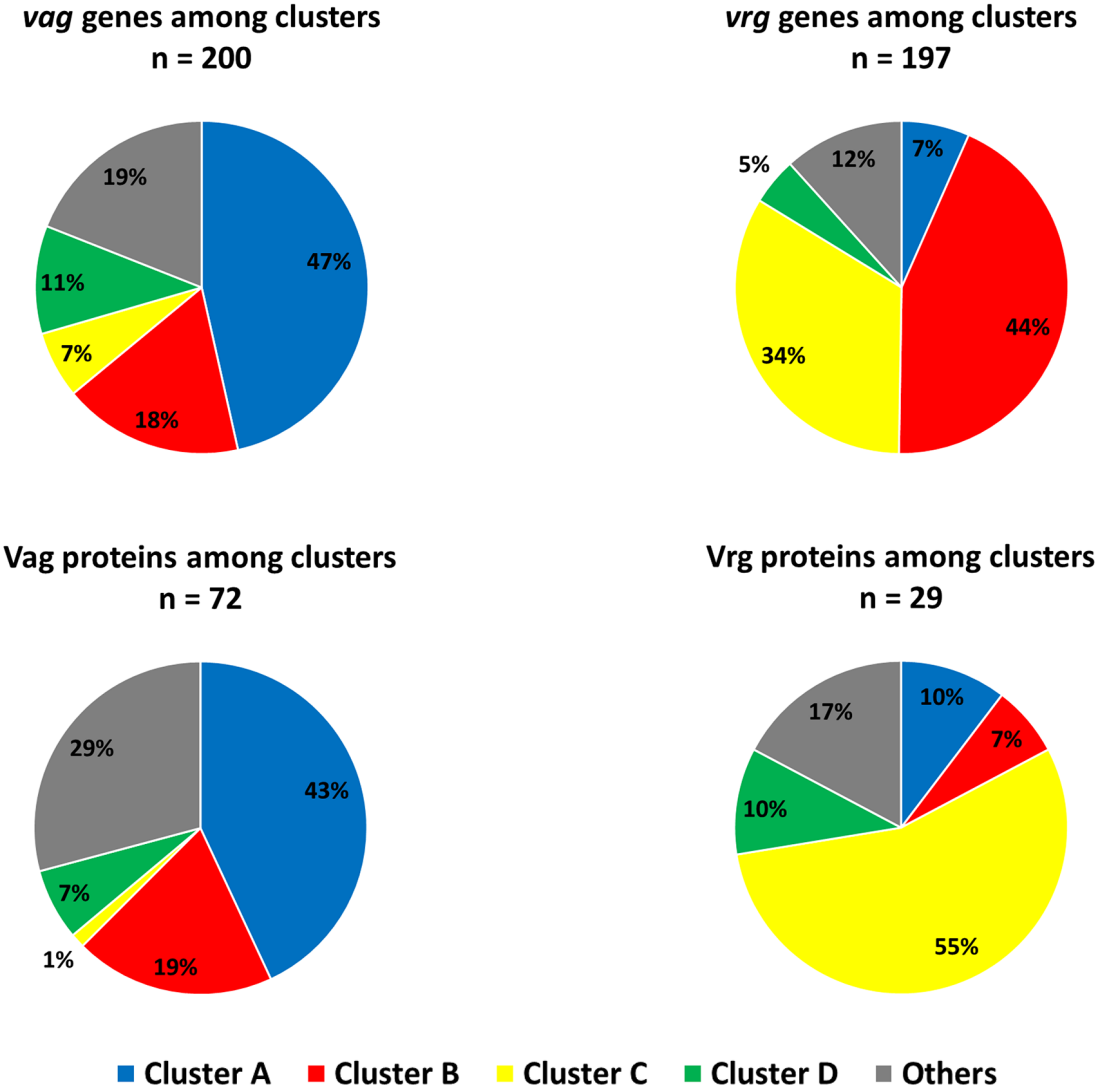
*vags* and *vrgs* transcripts and their associated proteins distribution among main clusters in transcriptomic and proteomic (Figures 4A,B) during *Bordetella pertussis* Tohama I culture. Based on 207 *vags* and 235 *vrgs* identified in recent studies (Moon et al., 2017; Coutte et al., 2020). “*n*=” corresponds to the number of genes/proteins clustered in our data among *vags* or *vrgs*.

The proportions of *vags* and *vrgs* and their associated proteins in each main clusters (Figures 4A,B) were next investigated. For this purpose, a list of 207 *vags* (log_2_FC < −2) *and 235 vrgs* (log_2_FC > 2; Supplementary Tables 11, 12) was selected from the literature (Moon et al., 2017; Coutte et al., 2020). Among those *vags*, 200 genes (97%), and 72 (35%) of their associated proteins were assigned in one of the cluster of our study (Figure 9). Surprisingly, the *vags* were not co-clustered. Indeed, 47, 18, 7, and 11% belong to the cluster A–D, respectively. Similar observation was highlighted with *vags* associated proteins, with 43, 19, 1, and 7%, respectively, associated with the cluster A–D, with 76% of consistency between proteomic and transcriptomic. Among the *vrgs*, 197 genes (84%) and 29 (12%) of their associated proteins passed the filtration steps. Similarly to the *vags*, the *vrgs* were spread among several clusters (Figure 9).

### Similar gene expression profiles between *vags* devoid of BvgA-binding site and putative regulator

**Figure 10:**
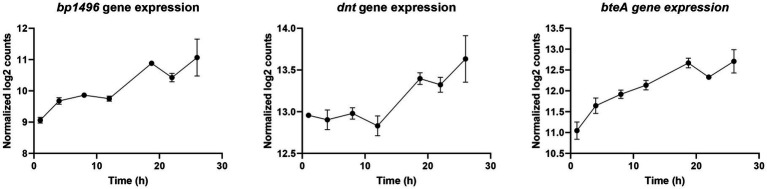
Longitudinal expression profiles of *bp1496*, *dnt*, and *bteA* genes during *Bordetella pertussis* Tohama I culture.

Recently, Coutte et al. showed with RNA-Seq/ChIP-Seq experiments that 16 *vags*, including the dermonecrotic toxin gene (*dnt* = *bp3439*), do not bear a BvgA-binding site (Coutte et al., 2020). They suggested that *vags* without BvgA-binding site may be indirectly regulated by BvgAS *via* 20 putative intermediate regulators (Coutte et al., 2020). In our study, to discover new virulence regulation mechanisms in *B*. *pertussis*, the longitudinal gene expression profiles of these 20 putative regulators were compared to the profile of 16 recently described *vags* without BvgA-binding sites (Supplementary Table 1; Coutte et al., 2020). Interestingly, *bp*1496, *dnt* (*bp3439*), and *bteA* (*bp0500*) co-clustered in cluster B and thus show similar transcription profiles (Figure 10). Of note, BP1496 protein was not detectable in our experimental conditions.

At the genomic level, *bp1496* and *bp1495* genes are organized in operon (data not shown), which were previously described as vags (Coutte et al., 2020). Homology detection and structure prediction (HHpred; Zimmermann et al., 2018) analysis suggested that BP1495 contains a Histidine phosphotransfer domain (Hpt) and BP1496 was predicted to contain a response-regulator (RR) and a helix-turn-helix (HTH) DNA-binding domains.

### Putative cysteine starvation between 4 and 8 h of culture

Apart of our cluster analysis, we next focused the interpretation of our longitudinal dataset, on the cysteine metabolic pathway. Due to a lack of technical sensitivity, our analysis of the extracellular medium using NMR did not reveal the presence of cysteine. We thus investigated the sulfur uptake at the transcriptomic level. Transcription profiles of cysteine ABC transporter genes (*bp1362*, *bp1363*, and *bp1364*) showed an upregulation (log_2_FC = +2.9 to log_2_FC = +4.0) between 4 and 8 h after inoculation. A differential gene expression analysis, between 4 and 8 h of culture, revealed 19 downregulated (log_2_FC < −1, *p*-value < 0.05) and 62 upregulated genes (log_2_FC > 1, *p*-value < 0.05; Supplementary Table 5). Among significantly downregulated genes, genes encoding enzymes requiring cysteine or reduced glutathione, such as *bp2871*, *bp3007*, *bp3011*, and *bp3250*, were identified (Table 1). Conversely, cystine, methionine (*metN = bp2816*, *bp2817*, and *bp2818*), molybdate (*modA* = *bp3095*, *modB* = *bp3094*, and *modC* = *bp3093*), and sulfate (*sbp* = *bp0966*, *cysT = bp0967*, *cysW = bp0968*, and *cysA = bp0969*). ABC transporter genes were upregulated. Genes encoding enzymes for the transformation of sulfite in sulfate through the 3′-phosphoadenylyl sulfate (PAPS) intermediate (*bp0970* and *cysD = bp0970A*) were also upregulated between 4 and 8 h of culture (Parkhill et al., 2003; National Center for Biotechnology Information, 2022). However, according to KEGG database, this pathway is not present in *B*. *pertussis* genome (Kyoto Encyclopedia of Genes and Genomes, 2022).

**Table 1:** Main differentially expressed genes involved in sulfur metabolism between 4 and 8 h of culture.

Old locus tag	Gene name	Gene product	Log_2_ FC	Adjusted *p*-value	Mean expression (log_2_ counts)
*bp3095*	modA	Molybdate ABC transporter substrate-binding protein	**5**.**31**	7.14E-14	8.65
*bp1363*		Amino acid ABC transporter permease	**3**.**99**	6.05E-15	6.97
*bp3097*		CGNR zinc finger domain-containing protein	**3**.**88**	1.68E-10	7.15
*bp1364*		Amino acid ABC transporter substrate-binding protein	**3**.**57**	7.07E-14	9.63
*bp3432*	cysI	Nitrite/sulfite reductase (EC 1.8.7.1)	**3**.**41**	6.65E-15	8.73
*bp3094*	modB	Molybdate ABC transporter permease subunit	**3**.**21**	7.59E-14	6.34
*bp3093*	modC	Molybdenum ABC transporter ATP-binding protein (EC 7.3.2.5)	**2**.**92**	4.86E-14	6.97
*bp1362*		Amino acid ABC transporter ATP-binding protein	**2**.**85**	6.03E-12	6.47
*bp0969*	cysA	Sulfate ABC transporter ATP-binding protein (EC 7.3.2.3)	**2**.**41**	1.29E-04	1.38
*bp0966*	sbp	Sulfate ABC transporter substrate-binding protein	**2**.**28**	2.18E-09	4.72
*bp0967*	cysT	Sulfate ABC transporter permease subunit CysT	**2**.**14**	5.58E-05	2.40
*bp2817*		ABC transporter permease	**2**.**13**	1.99E-08	6.86
*bp2818*		MetQ/NlpA family ABC transporter substrate-binding protein	**2**.**08**	2.65E-09	7.72
*bp2816*	metN	Methionine import ATP-binding protein MetN (EC 7.4.2.11)	**1**.**97**	8.21E-07	7.60
*bp0970A*	cysD	sulfate adenylyltransferase subunit CysD	**1**.**82**	6.17E-05	3.34
*bp0968*	cysW	Sulfate ABC transporter permease subunit CysW	**1**.**70**	1.47E-04	1.72
*bp0970*		phosphoadenylyl-sulfate reductase	**1**.**70**	2.70E-04	1.71
*bp3250*		Gamma-glutamylcyclotransferase (EC 4.3.2.7)	**−0**.**98**	1.27E-04	5.33
*bp2871*		Cysteine dioxygenase family protein	**−1**.**71**	7.87E-02	3.08
*bp2808*		YeiH family putative sulfate export transporter	**−1**.**73**	1.46E-08	4.75
*bp3011*		Cysteine dioxygenase family protein	**−2**.**21**	1.27E-08	6.08
*bp3007*		Gamma-glutamylcyclotransferase (EC 4.3.2.7)	**−2**.**21**	2.50E-07	5.68

Molybdate is precursor of molybdenum, which is an essential cofactor of enzymes such as the nitrite/sulfate reductases (Zhong et al., 2020; Kyoto Encyclopedia of Genes and Genomes, 2022). In *Escherichia coli*, the Nitrate/sulfate reductase CysI (homologs of CysI/BP3432 according to KEGG) is known to catalyze the transformation of sulfite in sulfide (Ostrowski et al., 1989; Kyoto Encyclopedia of Genes and Genomes, 2022). Interestingly, *cysI* (*bp3432*) gene was also upregulated between 4 and 8 h. In a subsequent enzymatic reaction of cysteine metabolic pathway, the combination of sulfide with acetyl-serine leads to the formation of cysteine and acetate. According to the KEGG database, no putative homologous gene that may encode an enzyme able to catalyze this reaction is present in *B*. *pertussis* genome.

In agreement with transcriptomic data (although with smaller amplitude), similar expression profiles were revealed by comparative proteomic analyses of samples collected between 8 and 12 h. Interestingly, this starvation was concomitant with the starvation of ascorbic acid in the extracellular medium (Supplementary Table 4; Supplementary Figure 5).

## Discussion and conclusion

In this work, we present the first longitudinal multi-omics investigation of *B*. *pertussis* under small-scale culture in batch mode using four different omics, subsequently analyzed with Gaussian processes, longitudinal clustering and pathway enrichment methods associated to longitudinal vaccine antigen quantification. This approach was deployed in order to gain insight into the physiology of fermenting *B*. *pertussis* in chemically defined modified SS-medium.

Through the addition of magnesium sulfate in culture media, it is well-known that sulfate is a modulator of virulence factors in of *B*. *pertussis* Tohama I strain (Lacey, 1960; Moon et al., 2017; Coutte et al., 2020). In classical media using cysteine as a source of sulfate, it is believed that sulfate progressively accumulates as a result of cysteine catabolism which in return downregulates the production of PT (Bogdan et al., 2001). In absence of cysteine, the use of inorganic thiosulfate as the sole sulfur source (that is consumed during the growth) is reported to lead to a > 2-fold improvement in PT production (Branco dos Santos et al., 2017). These evidences stress out the central role of sulfur in *B*. *pertussis* PT production. From this perspective, our study shows that between 4 and 8 h numerous genes encoding enzymes involved in the cysteine and glutathione catabolism are markedly down regulated while sulfur uptake genes are highly increased. Similar transcriptional regulation events were previously reported in *Lactococcus lactis* upon cysteine starvation (Sperandio et al., 2005). From the longitudinal data collected in our study, we thus extrapolate that *B*. *pertussis* may be exposed to cysteine starvation between 4 and 8 h of culture. This hypothesis is consistent with a previous report showing an up regulation of *B*. *pertussis* sulfur metabolism and sulfate/amino acid ABC transporter genes in THIJS medium (Van De Waterbeemd et al., 2009). Simultaneously to cysteine starvation, we unraveled that extracellular ascorbic and dehydroascorbic acids are rapidly consumed. Taking into account that ascorbic acid is known to be a potent reducing agent, the extracellular culture medium may then rapidly lose its reducing power during culture (Goto et al., 2020). This may cause the oxidation of cysteine into cystine and reduce the source of sulfur. However, despite its richness in cysteine residues, the slope of specific total PT production did not vary significantly between 4 and 8 h of culture. Thereby, *B*. *pertussis* may need to use different sulfur sources during the course of a bioreactor culture. One hypothesis is that the cysteine metabolization in modified SS-medium may not actually modulate *B*. *pertussis* virulence. Alternatively, the thiosulfate may be less sensitive to oxidation after an ascorbic acid starvation, avoiding a switch of sulfur source at the beginning of the culture. Based on the results of our study, several modifications of the modified SS-medium composition may be proposed. In agreement with the literature, such improvement may include the replacement of cysteine by thiosulfate (Branco dos Santos et al., 2017). An alternative adjustment of the modified SS-medium composition may also include an increase of ascorbate concentration which would delay oxidation of cysteine and potentially lead to an increase of vaccine antigen production and particularly, PT production.

Our study reveals a decrease of *B*. *pertussis* growth rate impacting the total biomass after 18 h 45 min of culture. This decrease, suggesting a reduce access to nutrients, is associated with an increase of specific total FHA production (following a sharp reduction of specific production between 1 and 12 h of culture). This growth decrease is also as associated with a reduction of specific total PRN and Fim2 productions but also with a reduction of the specific total PT production speed after 18 h 45 min of culture. These ELISA based quantification assays are in agreement with omics analyses showing that the transcripts of *dsbA*, *fhaC*, *prn*, and *fimD* genes are downregulated and that the Ptx, Prn, and FimD protein expression are negatively impacted after 18 h 45 min of culture.

Interestingly, the timing of these observations is synchronized with the expression profiles of specific longitudinal clusters of genes and proteins. The functional analysis of these specific clusters is consistent with the simultaneous and total consumption of triglycerides in our experimental conditions. Taking into account that these triglycerides are known to be a major energy stock for other bacterial species (Alvarez and Steinbüchel, 2002), we thus speculate that *B*. *pertussis* cells are under starvation for this endogenous source of energy once the triglycerides stock is fully consumed. This hypothesis is consistent with the subsequent accumulation of extracellular metabolites such as acetoacetate, hydroxybutyrate, and succinate observed from 18 h 45 min to 22 h. Indeed, hydroxybutyrate, and acetoacetate are degradation products of Poly-hydroxy-butyrate (PHB), which similar to triglycerides, is used as energy stock (Thalen et al., 1999). Interestingly, the accumulation and degradation of PHB were previously observed with the *B*. *pertussis* 509 strain grown in modified SS-medium although the mechanism triggering the degradation remains unknown (Thalen et al., 1999). Here, our study suggests that such PHB degradation is consecutive to proline starvation. Additional multi-omics investigations including intracellular metabolomic analyses paired with a specific PHB quantification method in presence of high concentration of proline will be necessary to confirm this hypothesis. Defenses against oxidative stress are reported to be deployed during starvation in bacteria (McDougald et al., 2002). Interestingly, PHB is also known to be mobilized when bacteria are exposed to oxidative stress (Müller-Santos et al., 2021). Hence, in *B*. *pertussis*, such response to oxidative stress is consistent with the production OxPE and the marked increase of superoxide dismutase gene (*sodA*) expression observed between mid to late exponential phases reported in our study and in the literature (Zavatti et al., 2020). Altogether, our data strongly suggest that during culture, *B*. *pertussis* is exposed to nutrient reductions and probably starvations by early exponential phase and uses alternate metabolisms of triglycerides and PHB as a source of energy.

A response of *B*. *pertussis* to nutrient reduction during the course of culture is also consistent with our data revealing proline starvation and an internal energy stock consumption (Figures 6A,B). Such response to starvation is in agreement with the expression/synthesis patterns of genes and proteins involved in glutamate and proline metabolisms. Indeed, with the reduction of extracellular proline (Figure 6A), we speculate that *B*. *pertussis* increases its uptake of glutamate as suggested by the upregulation of ABC transporters after 18 h 45 min of culture (Figure 6B). This increase of glutamate uptake may compensate the scarcity of proline by providing external glutamate which, in return, is converted to α-ketoglutarate to feed the TCA cycle. However, when proline is available, cells obtain ATP/energy from the oxidation of proline and the subsequent reduction of 1 FAD and 1 NAD^+^ into FADH_2_ and NADH (Surber and Maloy, 1998). Under proline starvation, *B*. *pertussis* is thus limited in reducing power and energy production. In the meantime, *B*. *pertussis* is exposed to oxidative stress, which may result from proline starvation. Therefore, the observed proline starvation may trigger PHB degradation which in return supplies the central metabolism at the acetyl-CoA level (Figure 6B). The increasing concentration of acetyl-CoA leads to an increase of gluconeogenesis and TCA cycle activity and PHB temporarily becomes the main carbon sources of culture. Simultaneously, the catabolism of other carbon sources (*eg*. glutamate) is inhibited, inducing an intracellular accumulation of those metabolites and inhibiting ABC transporters activity. The metabolic physiology proposed here is sustained with pathways enrichment analyses results of clusters C and D. Once PHB is entirely degraded, inhibition of carbon sources catabolism disappears, activating ABC transporters. Taken together, our data suggest the establishment of three culture steps: (i) Glutamate and proline are the main carbon sources up to proline starvation, (ii) Proline starvation leads to internal energy stock metabolization, which takes over the metabolism, and (iii) PHB is entirely degraded, leading to a basal metabolism with glutamate as a main carbon source. This hypothesis is supported by the functional assignment and the trajectories of the clusters A and B (which may be explained by a proline starvation) and the trajectories of clusters C and D (which could reflect a transient metabolism).

In bacteria, the metabolism and the synthesis of virulence factors are known to be closely related events (Richardson, 2019; Panayidou et al., 2020). In fermenting *B*. *pertussis*, previous studies have unraveled that specific *in vitro* nutrient limitations can decrease *B*. *pertussis* virulence (Nakamura et al., 2006; Van De Waterbeemd et al., 2009). Taking into account the proline starvation and the metabolic switch observed during our longitudinal study, we next explored the impact of culture on virulence factors regulation and biosynthesis. In *B*. *pertussis*, the core regulon includes factors that are required for efficient infection and transmission (Streefland et al., 2007). In bioreactors, the expression level of the *B*. *pertussis* core regulon is known to be highly correlated to *in vitro* growth (Streefland et al., 2007). Here, 33 of the 56 virulence core regulon genes of *B*. *pertussis* are shared between clusters A and B. This suggests that the expression of the virulence core regulon genes might be impacted by proline starvation as previously described during both lactate and glutamate limitation (Van De Waterbeemd et al., 2009). Taken together, our results confirm that the molecular response to carbon sources limitation is associated to a decrease of most *B*. *pertussis* virulence factor synthesis during bioreactor cultures. Thereby, a higher initial concentration of proline in the culture medium or a bolus of proline along the culture might prevent this metabolomic switch, maintain the growth and increase antigen production. Next, the main virulence regulators and virulence activated (*vag*) and repressed (*vrg*) genes were investigated in regard to the longitudinal clusters of genes and proteins of our study (Moon et al., 2017; Coutte et al., 2020). Interestingly, gene encoding *vags* and their associated proteins (including BvgASR) are not co-clustered and present heterogenous longitudinal expression patterns during culture. This finding confirms that other regulator(s) may be involved in the transcriptional regulation of *vags* (i.e *vags* devoid of *BvgA* binding site) in addition of BvgAS in our experimental conditions (Gestal et al., 2019; Coutte et al., 2020).

**Figure 11:**
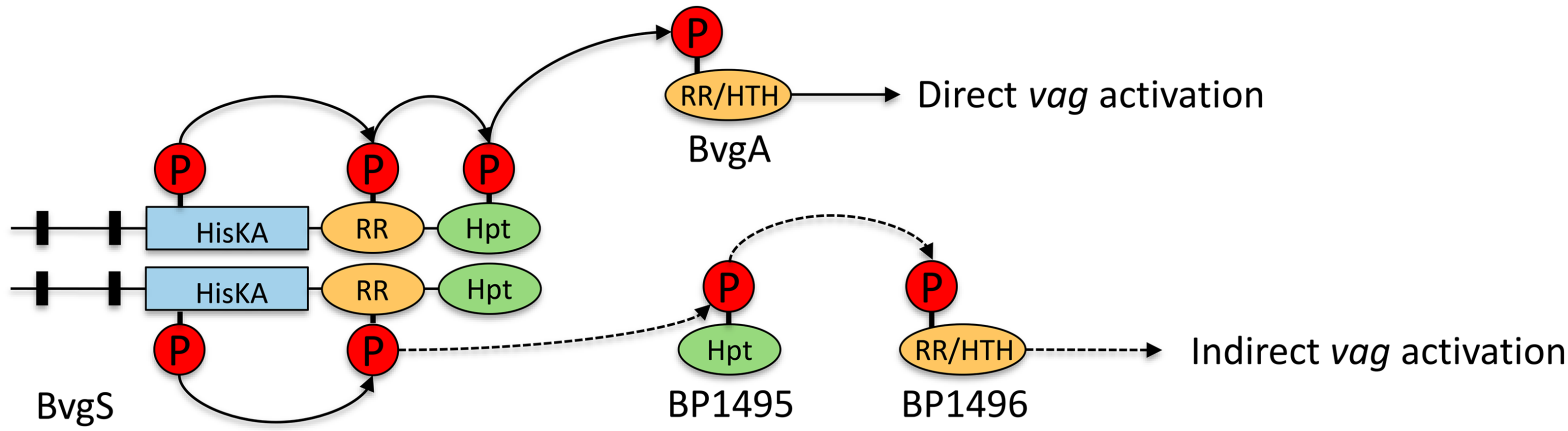
Scheme of putative indirect BvgA regulation on some *vags* such as *dnt* and *bteA* in *Bordetella pertussis*. Full arrows represent known phosphorylation pathways, dotted arrows represent hypothetical phosphorylation pathways. BvgS contains a Histidine-kinase (HisKA), a receiver (RR), and a Histidine phosphotransfer (Hpt) domains. BvgA is composed of a RR and an helix-turn-helix DNA-binding domain (HTH) (Dupré et al., 2015). A Hpt domain was predicted in BP1495 protein, a RR and a HTH domains were predicted in the BP1496 protein.

To explore this hypothesis, we compared the longitudinal transcriptional profiles of recently identified putative regulators with the transcriptional profiles of *vags* without BvgA-binding site (Coutte et al., 2020). Interestingly, among the genes of cluster B, the operonic genes *bp1496* (encoding a putative RR/HTH domain protein) and *bp1495* (encoding a putative Hpt domain protein) showed transcription trajectories that are similar to *dnt* and *bteA*. Thereby, we extrapolate that upon phosphorylation of BvgS RR domain a phosphate may be transferred to the Hpt domains of either BvgS or BP1495 and subsequently to BP1496. This phosphorylation cascade BP1495/ BP1496 may explain the indirect activation by BvgAS of the transcription of the 16 *vags* (including *dnt* and *bteA*) devoid of BvgA-binding site (Figure 11). Additional experiments using Δ*bp1495*/Δ*bp1496 B*. *pertussis* mutants and/or CHIPseq analyses will be necessary to experimentally confirm the role of this putative two-component system. From a vaccine manufacturing perspective, such data will be of primary importance since DNT is considered as an impurity in vaccine due to its high toxicity and its poor immunogenicity (Edwards and Decker, 2018).

In conclusion, this work using a longitudinal multi-omics approach combined to a vaccine antigen quantification provides new insights into the physiology of *Bordetella pertussis* Tohama I model in small-scale bioreactor cultures. Such longitudinal multi-omics analysis performed with a reference *B*. *pertussis* strain represents solid foundations toward the incremental optimization of vaccine antigen production with *B*. *pertussis* industrial strains. Moreover, in the future, with the addition of OMICs data obtained in conditions mimicking a low yield of vaccine antigen syntheses, these data may also constitute a reference to define monitoring molecular signatures that are predictive of batch failures. Associated to corresponding corrective counter measures, such predictive biomarkers may also contribute to pertussis vaccine manufacturing robustness and consistency.

## Data availability statement

The original contributions presented in the study are publicly available. RNA-seq data are available on SRA or Gene Expression Omnibus (GEO) (https://www.ncbi.nlm.nih.gov/geo/) with identifier GSE212531. The mass spectrometry proteomic data are available on Proteome Xchange repository (http://proteomecentral.proteomexchange.org/cgi/GetDataset) with identifier PXD039615. The mass spectrometry lipidomic data and the NMR extracellular metabolomic data have been deposited to the Metabolights (https://www.ebi.ac.uk/metabolights/) with identifier MTBLS5762.

## Author contributions

PA, JB, CM, NA-B, VC, MI, RP, JA-G, V-DT, MS, AB, DG, EA, GR-M, and CG contributed to manuscript redaction and to the design of study. PA contributed to culture process, culture QC, samples generation, ELISA, RNA-Seq, proteomic, and lipidomic and metabolomic experiments, analyzed metabolomic data, and interpreted omic data. EA was responsible of the project coordination at Sanofi Pasteur. JB carried out the biostatistic analyses of omic data. CM was responsible of the project coordination at BIOASTER. NA-B carried out bioinformatic analyses of RNA-Seq data. VC was responsible of RNA-Seq experiments. MI was responsible of culture and contributed to culture processes. RP was responsible of ELISA analyses. JA-G was responsible of bioinformatic analyses for proteomic and lipidomic data. V-DT carried out the bioinformatic analyses of proteomic and lipidomic data. MS contributed to lipidomic analysis. AB contributed to metabolomic analyses. MI, DG, EA, GR-M, and CG bring us their expertise of *Bordetella pertussis* physiology. All authors contributed to the article and approved the submitted version.

## Funding

Funding for this work was provided by Sanofi. Additional support came from BIOASTER technology research institute.

## Conflict of interest

PA, NA-B, VC, MI, RP, DG, EA, and GR-M were employed by Sanofi.

The remaining authors declare that the research was conducted in the absence of any commercial or financial relationships that could be construed as a potential conflict of interest.

## Publisher’s note

All claims expressed in this article are solely those of the authors and do not necessarily represent those of their affiliated organizations, or those of the publisher, the editors and the reviewers. Any product that may be evaluated in this article, or claim that may be made by its manufacturer, is not guaranteed or endorsed by the publisher.
