# Supplementary material for: Deep longitudinal multi-omics analysis of Bordetella pertussis cultivated in bioreactors highlights medium starvations and transitory metabolisms, associated to vaccine antigen biosynthesis variations and global virulence regulation
Source: Front Microbiol. 2023 Feb 14;14:1036386. doi: 10.3389/fmicb.2023.1036386 (PMC9976334; doi:10.3389/fmicb.2023.1036386)
Supplement: Supplementary file 1 [file Data_Sheet_1.PDF]

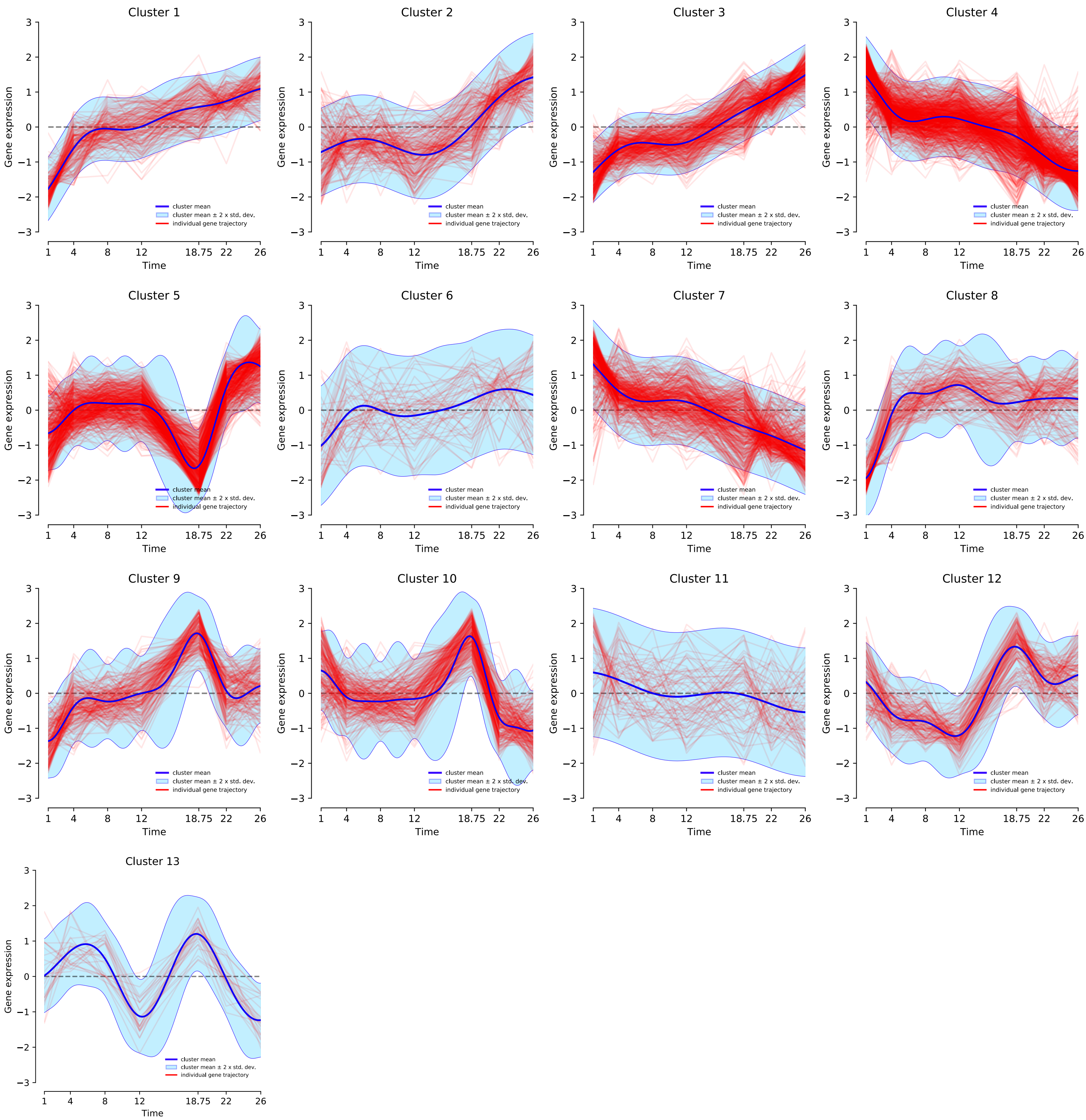

Supplementary Figure 1: Longitudinal clusters of transcriptomic data of *B. pertussis* culture. X-axis correspond to the time and Y-axis correspond to the gene expression. Blue line corresponds to the cluster mean, light blue interval corresponds to the cluster mean ± 2 x standard deviation and red line corresponds to the individual transcript trajectories.
