# Supplementary material for: Deep longitudinal multi-omics analysis of Bordetella pertussis cultivated in bioreactors highlights medium starvations and transitory metabolisms, associated to vaccine antigen biosynthesis variations and global virulence regulation
Source: Front Microbiol. 2023 Feb 14;14:1036386. doi: 10.3389/fmicb.2023.1036386 (PMC9976334; doi:10.3389/fmicb.2023.1036386)
Supplement: Supplementary file 5 [file Data_Sheet_5.PDF]

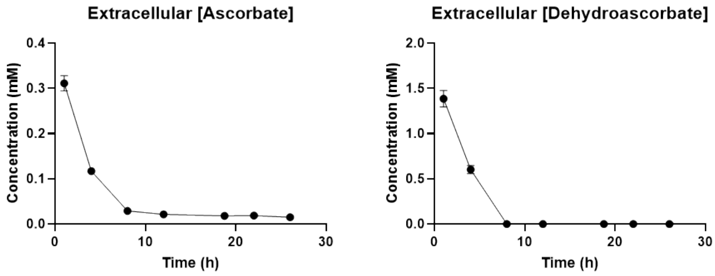

Supplementary Figure 5: Ascorbate and dehydroascorbate concentrations in the growth medium during *B. pertussis* Tohama I culture. Concentrations of metabolites (mM) were monitored by NMR.
